# Baseline Characterization of the Gut Microbiota of Field and Colony Populations of *Phlebotomus tobbi* and Preliminary Assessment of the Anti-Leishmanial Activity of Cultivable Bacteria

**DOI:** 10.3390/pathogens15070658

**Published:** 2026-06-23

**Authors:** Mehmet Karakuş, Ayda Yılmaz, Mert Okbay, Metin Pekağırbaş, Ozge Erisoz Kasap

**Affiliations:** 1 Department of Medical Microbiology, Hamidiye Faculty of Medicine, University of Health Sciences, Istanbul 34668, Türkiye; 2 VERG Laboratories, Department of Biology, Faculty of Science, Hacettepe University, Beytepe, Ankara 06800, Türkiye; aydayilmaz93@gmail.com (A.Y.); mert.okbay@uzh.ch (M.O.); erisoz@hacettepe.edu.tr (O.E.K.); 3 Department of Parasitology, Faculty of Veterinary Medicine, Aydın Adnan Menderes University, Aydın 09016, Türkiye; metin.pekagirbas@adu.edu.tr

**Keywords:** *Phlebotomus tobbi*, midgut microbiota, 16S rRNA sequencing, MALDI-TOF, leishmanicidal activity, *Leishmania infantum*, paratransgenesis

## Abstract

Sand fly midgut microbiota plays a critical role in shaping *Leishmania* development and vector competence, yet functional evidence from natural vector populations remains limited. In this study, sand flies were collected between 2020 and 2022 in Cukurova region, Türkiye to characterize the gut bacterial composition of *Phlebotomus tobbi* and evaluate the anti-leishmanial potential of cultivable isolates. A total of 1739 sand flies were captured (878 females, 861 males), of which *Ph. tobbi* was the predominant species (n = 1312). 16S rRNA amplicon sequencing (V4–V6) showed that the gut microbiota was dominated by Proteobacteria, with *Erwinia aphidicola*/*persicina* representing the most abundant species across all analyzed groups. Fourteen cultivable bacterial species were identified by MALDI-TOF MS, including *Serratia liquefaciens*, *Pantoea agglomerans*, and *Micrococcus luteus*. Functional XTT assays against *Leishmania infantum* promastigotes demonstrated variable inhibitory activity among isolates. The strongest leishmanicidal effects were observed with *S. liquefaciens* (32.3%) and *M. luteus* (28.8%). Morphological examination confirmed promastigote rounding and cell death in isolates showing >25% activity. These findings define the gut bacterial landscape of *Ph. tobbi* in an endemic region and identify bacterial taxa with in vitro anti-leishmanial activity, highlighting their potential for future microbiota-based or paratransgenic control strategies.

## 1. Introduction

*Phlebotomine* sand flies serve as vectors of several human diseases, including leishmaniasis, sand fly fever, and bartonellosis [1]. Among these, leishmaniasis remains a major public health problem in the Mediterranean Basin, largely due to high population density and sustained transmission [2]. Depending on the infecting *Leishmania* species and the host’s immune status, the disease manifests primarily in two clinical forms: cutaneous leishmaniasis (CL), which is typically self-limiting and endemic in 85 countries, and visceral leishmaniasis (VL), a potentially fatal form endemic in 74 countries [3]. Sand fly vectors acquire the parasite during blood feeding, ingesting amastigote forms that transform into infective metacyclic promastigotes within the midgut, a process known as metacyclogenesis. This critical developmental step is influenced not only by parasite genetics but also by the microbial environment of the sand fly gut [4,5].

Türkiye remains an endemic region for both VL and CL, reporting approximately 2000 CL cases and 40 VL cases annually [1]. Nine *Phlebotomus* species are either confirmed or suspected vectors of Old World leishmaniasis in the country, with the highest burden concentrated in Anatolia and the Mediterranean regions [6,7]. *Phlebotomus tobbi* is one of the proven vectors of *L. infantum* in the Mediterranean region of Türkiye, with distribution extending to Cyprus, Albania, Greece, and Iran [8,9]. Recent studies indicate that in the Çukurova region, *Ph. tobbi* serves as the sole vector for both human CL and canine leishmaniasis (CanL) and is common across all three major biogeographical regions of Türkiye [8]. Although several vector control studies have been conducted in the region, interventions have largely been restricted to chemical methods, with no approaches targeting parasite development within the vector itself [10].

The life cycle of *Leishmania* involves multiple differentiation events enabling adaptation to distinct host environments. Increasing evidence highlights the pivotal role of sand fly gut microbiota in shaping parasite development, modulating immunity, and ultimately influencing vectorial capacity [4]. Stable associations between sand flies and bacterial symbionts, often maintained through vertical transmission, suggest long-term co-evolutionary interactions. These bacteria can impact larval survival, adult fitness, and susceptibility to *Leishmania* infection [4,11,12]. Experimental studies further indicate that bacterial metabolites secreted during gut colonization significantly influence *Leishmania* growth, differentiation, and survival [12,13]. Geographic variation in bacterial composition has also been reported in sand fly populations, including those from Türkiye, suggesting that local microbial communities may shape transmission dynamics [14]. Collectively, these findings emphasize a complex tripartite interaction among vector, parasite, and microbiota, with direct implications for disease epidemiology [15,16].

Despite these advances, our understanding of sand fly-associated microbiota remains limited compared with other vector systems such as mosquitoes and triatomines. While paratransgenesis has been successfully applied in *Rhodococcus rhodnii* of *Rhodnius prolixus* against *Trypanosoma cruzi*, applications in sand flies are still scarce [17,18]. Establishing paratransgenesis in new ecological settings first requires detailed characterization of the bacterial diversity associated with target sand fly species. The success of such approaches depends on understanding the composition and functional roles of midgut microbiota, as well as their interactions with *Leishmania*. However, the lack of baseline data on bacterial diversity and functional capacity continues to constrain the development of microbiota-based control strategies in leishmaniasis-endemic regions.

Türkiye harbors diverse ecological zones with multiple sand fly species and parasite lineages, yet no studies have systematically examined how bacterial communities vary across sand fly populations or influence vector competence. Filling this gap is critical not only for clarifying regional epidemiology but also for identifying bacterial taxa that may serve as candidates for paratransgenic interventions [17,19,20].

In this study, we address these gaps by characterizing the bacterial communities associated with field-collected *Phlebotomus tobbi* populations from endemic regions of Türkiye and testing the leishmanicidal activity of selected bacterial isolates in vitro. By linking microbiota composition with functional assays, we aim to generate new insights into the role of sand fly-associated bacteria in parasite transmission and to explore their potential for paratransgenic applications.

## 2. Materials and Methods

### 2.1. Study Area and Sand Fly Collection

Sand flies were collected from the Çukurova Basin in southern Türkiye, a region bordered by the Amanos Mountains to the east, the Western Taurus Mountains to the west, and the Mediterranean Sea to the south, covering an area of approximately 3150 km^2^. Sampling was conducted during the summers of 2020–2022 in four villages: Camili, Damyeri, Otluk, and Zerdali. Sites were selected based on suitability for sand fly breeding and resting, including animal shelters (barns, poultries) and peridomestic environments, as described previously [10,21]. CDC miniature light traps (10 traps were deployed at each village during each sampling night) were placed indoors, outdoors, and near vegetation, operating from dusk (~19:00) until dawn (~06:00). Captured sand flies were transported to the laboratory for morphological identification and sorting. Species identification was performed using previously published keys [22,23].

### 2.2. Establishment of Laboratory Colonies

A colony of *Phlebotomus tobbi* was established from field-collected specimens and maintained at the VERG Laboratories, Department of Biology, Hacettepe University, following established procedures [24,25]. Both male and female progeny from the laboratory-reared F1 generation obtained from parental females were used in the bacterial colonization and vertical transmission experiments.

### 2.3. Midgut Dissections and Bacterial Diversity

Sand flies were sorted by sex and physiological status prior to dissection. To minimize contamination, specimens were washed in antimicrobial solution (Gibco™ Pen-Strep, NY, USA 10,000 U/mL) before processing. Midguts were dissected under sterile conditions as described previously, and each was examined microscopically to detect natural *Leishmania* infections [26]. Dissected gut material was divided into two portions: one was inoculated on various culture media (TSA, PCA, MHB, NB), while the other was preserved for molecular analysis of bacterial diversity.

Cultivable bacterial isolates were identified using MALDI-TOF mass spectrometry. For culture-independent analysis, sand flies were pooled by sex and collection site (≤10 individuals per pool) and preserved in RNA/DNA Shield (Zymo Research, FL, USA) [27]. DNA was extracted using the ZymoBIOMICS DNA Miniprep Kit (Zymo Research) according to the manufacturer’s instructions. The V4–V6 regions of the bacterial 16S rRNA gene were amplified and sequenced by next-generation sequencing (NGS). Taxonomic classification and relative abundance profiles were generated using Geneious v9 (Biomatters, NY, USA). Library preparation and sequencing were performed commercially by Zymo Research.

### 2.4. Leishmanicidal Activity of Bacterial Isolates

The leishmanicidal activity of bacterial isolates was evaluated using the XTT viability assay. *Leishmania infantum* (MHOM/TN/80/IPT-1) promastigotes were thawed from cryopreservation, cultured in GIBCO RMPI-1640 NY, USA medium supplemented with 15% fetal bovine serum (FBS), and maintained until reaching the plateau growth phase, which was confirmed by Thoma chamber counts. Bacterial isolates were prepared at different CFU/mL concentrations in RPMI-1640 and co-cultured with promastigotes (1 × 10^6^/mL) in 96-well plates (100 µL/well each). Plates were incubated at 24 °C for 48 h, after which parasite viability was assessed. Pentostam was used as the positive control and RPMI-1640 medium as the negative control. All experimental conditions, including each bacterial isolate and control group, were prepared and tested in triplicate independent replicates. XTT normalization formula [(%) = [100 × (sample absorbance)/(control absorbance)] applied as described previously [28]. Mortality values were calculated as the mean of three replicate measurements, and variability among replicates was expressed as standard deviation (SD), which was represented in the graphical analyses.

### 2.5. Molecular Surveillance of Leishmania Infections in Field-Collected Sand Flies

Female *Ph. tobbi* specimens were pooled (5–10 individuals per pool) in ZR Bashing Bead^TM^ tubes (Zymo Research). Positive control DNA was obtained from reference isolates (MHOM/TN/80/IPT-1), while male sand flies served as negative controls. Pools were homogenized in a Magna Lyser (Roche, Mannheim, Germany) at 7000 g for 50 s, resuspended in 200 µL Qiagen tissue lysis buffer, and incubated overnight at 56 °C. DNA was extracted using the DNeasy Blood & Tissue Kit (Qiagen, Hilden, Germany) and eluted in 50 µL of buffer. *Leishmania* DNA was detected using kinetoplast DNA (kDNA)-specific primers (JW11/JW12) with the SYBR Green I Master Kit (Roche, Mannheim, Germany), following the protocol described recently [29].

## 3. Results

### 3.1. Sand Fly Collections and Species Composition

A total of 1739 sand flies were collected from four field campaigns conducted in the İmamoğlu and Kozan districts (Adana Province) between 2020–2022. Specimens comprised 878 females and 861 males. Specimen identification revealed dominance of *Ph. tobbi* (n = 1312; 75.5%), followed by *Ph. papatasi* (n = 338; 19.4%), *Sergentomyia dentata* (n = 44; 2.5%), *Ph. major* s.l. (n = 23; 1.3%), *Ph. perfiliewi* s.l. (n = 8; 0.5%), and *Ph. sergenti* (n = 8; 0.5%). Two specimens of *Adlerius* sp. were also recorded. Sampling sites were located at 176–214 m elevation, mainly within enclosed animal shelters with sheep, goats, dogs, and cattle as potential blood sources (Table 1).

**Table 1:** Species composition of sand flies collected from İmamoğlu and Kozan districts (2020–2022).

Species	Female (n)	Male (n)	Total (n)	% of Total
*Phlebotomus tobbi*	660	652	1312	75.5%
*Ph. papatasi*	176	162	338	19.4%
*Sergentomyia dentata*	22	22	44	2.5%
*Ph. major* s.l.	12	11	23	1.3%
*Ph. perfiliewi* s.l.	4	4	8	0.5%
*Ph. sergenti*	4	4	8	0.5%
*Adlerius* sp.	-	2	2	0.1%

### 3.2. Surveillance of Leishmania Infection

Midgut dissections were performed on all 878 female specimens. No promastigote infections were detected microscopically. Subsequently, molecular screening was conducted on 84 pools (67 *Ph. tobbi*, 17 *Ph. papatasi*) using kDNA-targeted real-time PCR, and no *Leishmania* DNA was detected.

### 3.3. Bacterial Diversity in Field and Colony Specimens

Metagenomic profiling of *Ph. tobbi* midguts revealed 15 bacterial taxa spanning *Actinobacteria*, *Firmicutes*, and *Proteobacteria*. Community composition varied by sand fly sex and feeding status. Field-collected females were dominated by *Erwinia aphidicola*/*persicina* (46.3%), *Serratia marcescens*/*nematodiphila* (20.8%), and *Ralstonia pickettii* (7.9%). Males showed higher prevalence of *Erwinia aphidicola*/*persicina* (63.6%) and *Pantoea ananatis* (13.9%). Blood-fed females were enriched for *Burkholderia* (*Paraburkholderia*) *fungorum* (18.5%), *Stenotrophomonas maltophilia* (14.9%), and *Serratia marcescens/nematodiphila* (17.4%).

Colony females were dominated by *Erwinia aphidicola/persicina* (41.4%) and *S. maltophilia* (30.1%), whereas colony-derived males showed dominance of *E. aphidicola/persicina* (46.3%) and *S. maltophilia* (23.4%) (Table 2). Alpha and beta-diversity analyses indicated reduced richness in colony populations compared to field specimens. Rare taxa (<0.1%) were excluded from graphical analyses to avoid distribution bias. All metagenomic data was presented as Supplementary File S1.

**Table 2:** Relative abundance of dominant bacterial taxa in *Ph. tobbi* midguts.

Bacterial Taxon *	Field Female	Field Male	Colony Female	Colony Male
*E. aphidicola/persicina*	46.3%	63.6%	41.4%	46.3%
*S. marcescens/nematodiphila*	20.8%	9.7%	5.6%	6.4%
*P. ananatis*	2.9%	13.9%	—	—
*B. fungorum*	18.5%	—	—	—
*S. maltophilia*	14.9%	—	30.1%	23.4%
*R. pickettii*	7.9%	4.7%	—	—

* Only taxa with ≥0.1% abundance in at least one group are shown.

### 3.4. Cultivable Bacteria and MALDI-TOF Identification

Field-derived isolates grown on TSA, PCA, MHA, and broth media were Gram-characterized and identified via MALDI-TOF. Fourteen species were confirmed: *Staphylococcus cohnii*, *Pantoea agglomerans*, *Bacillus simplex*, *Serratia liquefaciens*, *Micrococcus luteus*, *Leuconostoc mesenteroides*, *Enterococcus faecium*, *Bacillus pumilus*, *Streptococcus equinus*, *Bacillus clausii*, *Lactobacillus casei*, *Lactobacillus rhamnosus*, *Paenibacillus cookii*, and *Lactobacillus paracasei*.

### 3.5. Leishmanicidal Activity of Sellected Bacterial Isolates

**Figure 1:**
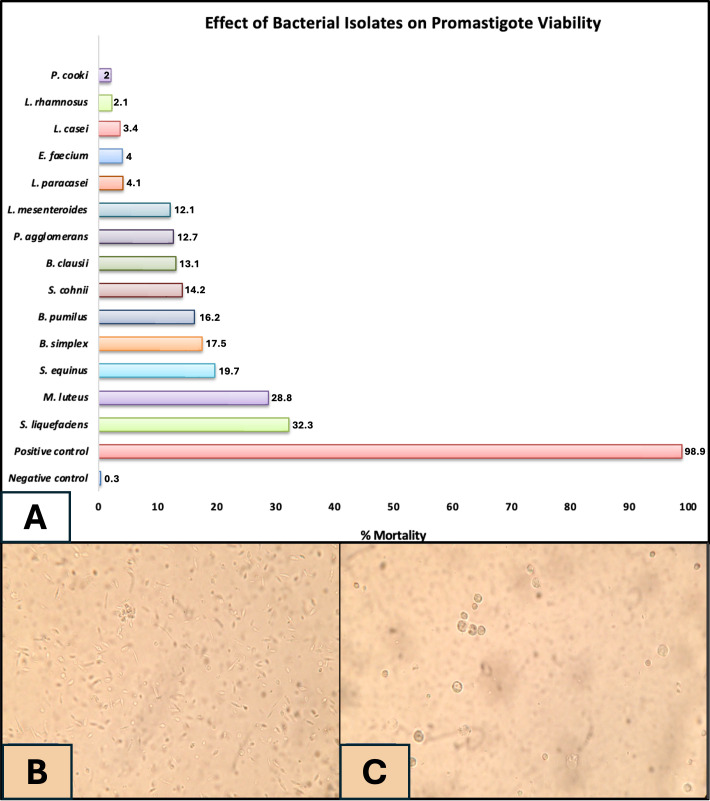
Leishmaniacidal effects of cultured bacterial isolates on *L. infantum* promastigotes (**A**) Percent mortality of *L. infantum* promastigotes after 24 h co-incubation with bacterial cultures (XTT assay). Mean ± SD of three independent experiments. (**B**) Control group promastigotes (**C**) Treatment group promastigotes (*Serratia liquefaciens*).

All cultured bacterial isolates were tested against *L. infantum* promastigotes using the XTT assay, and the results were expressed as the mean mortality percentages obtained from three independent replicate experiments. Mortality rates varied among species: *S. liquefaciens* (32.3%), *M. luteus* (28.8%), and *S. equinus* (19.7%) exhibited the highest inhibitory effects. Moderate activity was observed for *B. simplex* (17.5%), *B. pumilus* (16.2%), and *S. cohnii* (14.2%), whereas several isolates, including *E. faecium*, *L. casei*, *L. rhamnosus*, *P. cookii*, and *L. paracasei*, showed negligible activity (<5%). Variability among replicate measurements was represented in the graphical presentation, allowing visualization of reproducibility across triplicate assays. Morphological examination further confirmed promastigote rounding and cell death in isolates demonstrating >25% inhibitory activity (Figure 1).

## 4. Discussion

This study provides the first comprehensive characterization of the midgut microbiota of *Phlebotomus tobbi*, both in field-collected and laboratory-colonized populations, and evaluates the functional impact of bacterial isolates on *Leishmania infantum* promastigotes. To our knowledge, this is the first such study conducted in Türkiye to define the bacterial community composition of *Ph. tobbi* midguts using high-throughput 16S based sequencing in combination with cultivation-based approaches and functional assays. The findings therefore represent a critical baseline dataset that enhances our understanding of vector biology and the ecological determinants of leishmaniasis transmission.

One of the major questions of this study was to determine the midgut bacterial composition of field-collected *Ph. tobbi* specimens and their dynamics in such hyperendemic region for leishmaniasis. Sand fly-associated microbiota have been examined in several vector species, most notably *Ph. papatasi*, *Ph. argentipes*, and *Lutzomyia longipalpis*, and very different bacterial profiles were reported [30,31]. These data highlight that sand fly-associated microbiota is influenced not solely by the vector species but also by geographic determinants. Thus, vector microbiome studies should also include geographical determinants and should be evaluated in this context. Baseline microbiota characterizations are indispensable since they provide the foundation for hypothesis-driven research on host–microbe–parasite interactions and enable future longitudinal or intervention studies to contextualize their results. By establishing baseline microbial profiles for *Ph. tobbi* in such hyperendemic region, we believe that our study will support future paratransgenesis studies.

For Türkiye, the importance of this dataset is amplified by the country’s unique ecological setting. The Çukurova Basin, from which the specimens were collected, represents one of the most active transmission foci for both cutaneous and visceral leishmaniasis. The study area exhibits high *Leishmania* positivity, which is influenced by various ecological, social, and epidemiological factors [32]. Cross-sectional analysis revealed a CanL prevalence of 27.18% using IFAT and 41.74% through other diagnostic methods, indicating significant infection rates in these areas [21]. Understanding the microbiota of local sand fly populations is therefore not only an academic exercise but a step toward developing targeted strategies for leishmaniasis control in endemic foci. The data may also be extrapolated to neighboring Mediterranean and Middle Eastern regions, where *Ph. tobbi* co-occurs and contributes to transmission cycles.

An additional contribution of this study is the comparison between field-derived and laboratory-colonized *Ph. tobbi* populations. The gut microbiota of *Ph. tobbi* demonstrates notable differences between laboratory-reared colonies and wild populations. As also found in this study, previous researchers have likewise reported reduced bacterial richness, compared to the broader diversity observed in wild populations [26,33]. This reduction raises two important considerations: (i) colony-based experimental systems may underestimate the complexity of natural microbiota and thus provide an incomplete picture of vector–parasite interactions, and (ii) certain bacteria with potential functional relevance in parasite development or inhibition may be lost under laboratory conditions [34]. The persistence of *Erwinia* in both environments suggest strong host dependence, whereas the enrichment of *Stenotrophomonas maltophilia* in colonies may reflect adaptation to laboratory conditions [35]. These insights highlight the importance of validating laboratory findings against field-derived baselines.

Globally, the gut microbiota of sand flies is predominantly composed of *Proteobacteria* and *Firmicutes*, with *Proteobacteria* often representing the majority in wild populations [33]. This bacterial composition plays a crucial role in supporting the development of parasites within the sand fly gut lumen, where the presence of symbiotic and commensal bacteria influences parasite survival and maturation [34,36]. Studies also suggest that certain microbial symbionts may either enhance or inhibit activity, depending on the bacterial species present, further highlighting the microbiota’s role in vector competence [35]. These findings align with broader observations in other sand fly species, where the microbiota of wild populations displays greater richness and functional diversity compared to laboratory-reared counterparts. Such differences are important to consider in efforts to manipulate sand fly microbiota as part of integrated control strategies for leishmaniasis [36,37,38].

Across both field-collected and laboratory-reared populations of *Ph. tobbi*, *Erwinia aphidicola/persicina* emerged as the dominant bacterial taxon, accounting for up to 63% of sequences in males and more than 40% in females, indicating a stable and potentially well-adapted association between *Ph. tobbi* and *Erwinia* spp. This persistent dominance may reflect vertical transmission, efficient colonization of the sand fly gut, or strong ecological adaptation to the host intestinal environment. Although *Erwinia* is widely recognized as a plant-associated bacterium, accumulating evidence from other arthropod systems suggests that certain *Erwinia* species can support host nutrition by supplying essential amino acids and vitamins and may persist throughout metamorphic stages, highlighting their broader symbiotic potential [11,39,40]. In sand flies, however, the functional significance of this association remains unresolved. It is plausible that *Erwinia* contributes to nutrient provisioning, modulation of gut physiology, immune homeostasis, or competitive exclusion of other microbial taxa, all of which may indirectly influence vector competence. Given that gut-associated bacteria can affect parasite establishment by altering nutrient availability, immune responses, or facilitating attachment of *Leishmania* to the gut epithelium, the high abundance of *Erwinia* raises the possibility that it may either promote or inhibit parasite development depending on its ecological interactions within the gut microbiome [36,41]. Similar observations in related bacteria, such as *Delftia tsuruhatensis,* which can interfere with parasite colonization, further support the relevance of exploring these microbial interactions [42]. Taken together, the abundance of *Erwinia* in *Ph. tobbi* make it a compelling target for deeper functional studies and a possible candidate for microbiota-based vector control approaches, including paratransgenic manipulation or selective microbial disruption.

The second notable finding was the recurrent detection of *Serratia* species, particularly *Serratia marcescens/nematodiphila* in sequencing data and *Serratia liquefaciens* among cultivable isolates. *Serratia* has been repeatedly associated with inhibitory effects on several bacteria and a strong association with infection-driven midgut microbiota [35,43]. Our functional assays confirmed that *S. liquefaciens* exhibited the highest leishmanicidal activity, killing over 30% of promastigotes in vitro. This corroborates findings in other sand fly vectors, where *Serratia* spp. have been implicated in reducing parasite development through production of antimicrobial peptides, proteases, or secondary metabolites [44,45,46]. The consistent isolation of *Serratia* from *Ph. tobbi* and its demonstrated inhibitory capacity highlights it as possible candidate for paratransgenic interventions aimed at reducing *Leishmania* transmission. *Micrococcus luteus*, which showed nearly 29% mortality in our assays, has been reported to produce carotenoids and antimicrobial metabolites with broad inhibitory potential [47]. *Streptococcus equinus*, *Bacillus simplex*, and *Bacillus pumilus* showed intermediate leishmanicidal activity, aligning with prior observations that *Bacillus* spp. can generate lipopeptides and bacteriocins with antiprotozoal properties [46]. Conversely, lactic acid bacteria such as *Lactobacillus casei*, *L. rhamnosus*, and *L. mesenteroides* showed negligible activity, though their probiotic potential and influence on gut homeostasis cannot be discounted. Collectively, these findings underscore the taxonomic and functional heterogeneity of the *Ph. tobbi* microbiota and the necessity of evaluating both dominant and rare taxa for their role in shaping vector competence.

Our molecular surveillance did not detect *Leishmania* DNA in any of the 878 dissected females or 84 pooled samples tested. The results obtained in this study are surprising when compared with the high *Leishmania* positivity rates reported in previous studies conducted in the region, both in humans and reservoir hosts [7,48]. While this may reflect true low prevalence during the sampling period, it is also possible that infection rates in the İmamoğlu and Kozan districts are temporally variable.

Paratransgenic strategies have emerged as one of the most promising microbiota-based approaches for controlling vector-borne diseases by exploiting symbiotic or commensal bacteria naturally associated with insect vectors and engineering them to express molecules that interfere with pathogen development [17,49]. In sand flies, several bacterial taxa have already been proposed as suitable candidates for such interventions, including *Serratia* AS1, *Enterobacter cloacae*, *Bacillus subtilis*, and *Ochrobactrum* spp., all of which possess characteristics favorable for stable colonization, genetic manipulation, and persistence across developmental stages [18,20,44]. In this context, our demonstration that *Ph. tobbi*-derived bacteria, particularly *Serratia liquefaciens* and *Micrococcus luteus*, exert measurable leishmanicidal activity is especially significant. Their natural association with *Ph. tobbi* suggests that they could potentially be engineered to deliver anti-*Leishmania* effector molecules directly within the sand fly midgut, analogous to successful paratransgenic systems developed in triatomines targeting *T. cruzi* [50]. Beyond these bacterial candidates, *Wolbachia* represents another highly attractive yet underexplored avenue for sand fly control. Previous studies conducted in Türkiye have documented the presence of *Wolbachia* in sand fly populations, while infection rates can reach as high as 90% in certain taxa, the overall positivity rates are reported at approximately 16%, indicating that this endosymbiont is naturally established in local vector populations [51]. This relatively notable prevalence suggests that *Wolbachia*-based strategies, which have already demonstrated success in mosquito-borne disease control programs, may be an alternate for future sand fly-targeted biocontrol applications in the region [52,53,54].

From a public health perspective, baseline microbiota surveys such as this are invaluable. They not only advance fundamental science but also open applied avenues for sustainable vector control. Given the limitations of chemical insecticides and the lack of effective vaccines against leishmaniasis, microbiota-based strategies have emerged as a potentially valuable avenue for reducing transmission. The prominence of *Erwinia* and *Serratia* in *Ph. tobbi* populations across different conditions suggests these bacteria should be prioritized for functional and applied research.

## 5. Conclusions

In summary, this study delivers the characterization of *Ph. tobbi* midgut microbiota in a hyperendemic region of Türkiye and provides baseline data that are critical for understanding the ecology of this important vector. These findings reinforce the concept that vector competence is not determined by the parasite alone but is the outcome of a complex tripartite interaction between sand flies, parasites, and their associated microbiota. Building on this baseline, future research can explore microbiota manipulation and paratransgenesis as novel, sustainable tools in the fight against leishmaniasis.

## 6. Study Limitations

Despite the valuable results, several limitations must be acknowledged. First, microbiome analyses were based on pooled midgut samples rather than individual specimens, which may have masked individual-level variation and limited the assessment of intra-population microbial diversity. Consequently, the results primarily reflect dominant bacterial taxa and should be interpreted as a baseline descriptive characterization rather than a comprehensive ecological analysis. Second, due to the pooled sampling design and the limited number of sequencing groups, robust statistical comparisons of microbial community structure could not be performed. Third, although 16S rRNA amplicon sequencing provided valuable information on bacterial composition, the sequencing workflow was not specifically designed to investigate low-abundance taxa, and the exclusion of rare taxa may have resulted in the underrepresentation of less abundant community members. In addition, the functional evaluation of bacterial isolates was restricted to in vitro XTT assays using promastigote forms of *L. infantum.* Therefore, the observed anti-leishmanial activity should be considered preliminary, as no experiments were conducted using intracellular amastigotes, macrophage infection models, or natural vector-parasite interaction systems. Furthermore, laboratory infection experiments involving Leishmania-sand fly interactions could not be performed because of biosafety and laboratory containment restrictions. Consequently, the potential effects of the identified bacterial taxa on parasite development, transmission dynamics, and vector competence remain unresolved. Finally, although several bacterial isolates exhibited measurable anti-leishmanial activity, important characteristics required for evaluating their suitability as paratransgenic candidates, including stable gut colonization, transstadial transmission, long-term persistence, genetic manipulability, and in vivo efficacy, were not investigated. Therefore, conclusions regarding microbiota-based control strategies should be considered exploratory and require further experimental validation.

## Data Availability

The original contributions presented in this study are included in the article/supplementary material. Further inquiries can be directed to the corresponding author.

## Supplementary Materials

The following supporting information can be downloaded at: https://www.mdpi.com/article/10.3390/pathogens15070658/s1, File S1: Raw data of Microbiota Analyses.

## Abbreviations

The following abbreviations are used in this manuscript:

TSA	Tryptic Soy Agar
PCA	Plate Count Agar
MHB	Mueller–Hinton Broth
NB	Nutrient Broth
CL	Cutaneous Leishmaniasis
VL	Visceral Leishmaniasis
CanL	Canine Leishmaniasis
CDC	Centers for Disease Control and Prevention
MALDI-TOF	Matrix-Assisted Laser Desorption/Ionization Time-of-Flight
