# Supplementary material for: Baseline Characterization of the Gut Microbiota of Field and Colony Populations of Phlebotomus tobbi and Preliminary Assessment of the Anti-Leishmanial Activity of Cultivable Bacteria
Source: Pathogens. 2026 Jun 23;15(7):658. doi: 10.3390/pathogens15070658 (PMC13416292; doi:10.3390/pathogens15070658)
Supplement: Supplementary file 1 [file pathogens-15-00658-s001.zip › pathogens-4357168-File S1/Alpha_Diversity/Rarefaction_Plots/rarefaction_plots.html]

Rarefaction Curves


|  |  |  |  |
| --- | --- | --- | --- |
| **Select a Metric:** | PD\_whole\_tree chao1 fisher\_alpha observed\_species shannon simpson simpson\_e simpson\_reciprocal | **Select a Category:** | BarcodeSequence LinkerPrimerSequence SampleID |

  

**Show Categories:
 
All
None
Invert**

**Legend**

|  |  |  |  |
| --- | --- | --- | --- |
| ▶ |  | ■ | **NA** |
| ∟ |  | ◆ | **Patient1** |
| ∟ |  | ◆ | **Patient2** |
| ∟ |  | ◆ | **Patient3** |
| ∟ |  | ◆ | **Patient4** |
| ∟ |  | ◆ | **Patient5** |
| ∟ |  | ◆ | **Patient6** |
| ∟ |  | ◆ | **Patient7** |
| ∟ |  | ◆ | **Patient8** |
| ∟ |  | ◆ | **Patient9** |
| ∟ |  | ◆ | **Patient10** |
| ▶ |  | ■ | **GTGCCAGCMGCCGCGGTAA** |
| ∟ |  | ◆ | **Patient1** |
| ∟ |  | ◆ | **Patient2** |
| ∟ |  | ◆ | **Patient3** |
| ∟ |  | ◆ | **Patient4** |
| ∟ |  | ◆ | **Patient5** |
| ∟ |  | ◆ | **Patient6** |
| ∟ |  | ◆ | **Patient7** |
| ∟ |  | ◆ | **Patient8** |
| ∟ |  | ◆ | **Patient9** |
| ∟ |  | ◆ | **Patient10** |
| ▶ |  | ■ | **Patient1** |
| ∟ |  | ◆ | **Patient1** |
| ▶ |  | ■ | **Patient2** |
| ∟ |  | ◆ | **Patient2** |
| ▶ |  | ■ | **Patient3** |
| ∟ |  | ◆ | **Patient3** |
| ▶ |  | ■ | **Patient4** |
| ∟ |  | ◆ | **Patient4** |
| ▶ |  | ■ | **Patient5** |
| ∟ |  | ◆ | **Patient5** |
| ▶ |  | ■ | **Patient6** |
| ∟ |  | ◆ | **Patient6** |
| ▶ |  | ■ | **Patient7** |
| ∟ |  | ◆ | **Patient7** |
| ▶ |  | ■ | **Patient8** |
| ∟ |  | ◆ | **Patient8** |
| ▶ |  | ■ | **Patient9** |
| ∟ |  | ◆ | **Patient9** |
| ▶ |  | ■ | **Patient10** |
| ∟ |  | ◆ | **Patient10** |
| ▶ |  | ■ | **NA** |
| ∟ |  | ◆ | **Patient1** |
| ∟ |  | ◆ | **Patient2** |
| ∟ |  | ◆ | **Patient3** |
| ∟ |  | ◆ | **Patient4** |
| ∟ |  | ◆ | **Patient5** |
| ∟ |  | ◆ | **Patient6** |
| ∟ |  | ◆ | **Patient7** |
| ∟ |  | ◆ | **Patient8** |
| ∟ |  | ◆ | **Patient9** |
| ∟ |  | ◆ | **Patient10** |
| ▶ |  | ■ | **GTGCCAGCMGCCGCGGTAA** |
| ∟ |  | ◆ | **Patient1** |
| ∟ |  | ◆ | **Patient2** |
| ∟ |  | ◆ | **Patient3** |
| ∟ |  | ◆ | **Patient4** |
| ∟ |  | ◆ | **Patient5** |
| ∟ |  | ◆ | **Patient6** |
| ∟ |  | ◆ | **Patient7** |
| ∟ |  | ◆ | **Patient8** |
| ∟ |  | ◆ | **Patient9** |
| ∟ |  | ◆ | **Patient10** |
| ▶ |  | ■ | **Patient1** |
| ∟ |  | ◆ | **Patient1** |
| ▶ |  | ■ | **Patient2** |
| ∟ |  | ◆ | **Patient2** |
| ▶ |  | ■ | **Patient3** |
| ∟ |  | ◆ | **Patient3** |
| ▶ |  | ■ | **Patient4** |
| ∟ |  | ◆ | **Patient4** |
| ▶ |  | ■ | **Patient5** |
| ∟ |  | ◆ | **Patient5** |
| ▶ |  | ■ | **Patient6** |
| ∟ |  | ◆ | **Patient6** |
| ▶ |  | ■ | **Patient7** |
| ∟ |  | ◆ | **Patient7** |
| ▶ |  | ■ | **Patient8** |
| ∟ |  | ◆ | **Patient8** |
| ▶ |  | ■ | **Patient9** |
| ∟ |  | ◆ | **Patient9** |
| ▶ |  | ■ | **Patient10** |
| ∟ |  | ◆ | **Patient10** |
| ▶ |  | ■ | **NA** |
| ∟ |  | ◆ | **Patient1** |
| ∟ |  | ◆ | **Patient2** |
| ∟ |  | ◆ | **Patient3** |
| ∟ |  | ◆ | **Patient4** |
| ∟ |  | ◆ | **Patient5** |
| ∟ |  | ◆ | **Patient6** |
| ∟ |  | ◆ | **Patient7** |
| ∟ |  | ◆ | **Patient8** |
| ∟ |  | ◆ | **Patient9** |
| ∟ |  | ◆ | **Patient10** |
| ▶ |  | ■ | **GTGCCAGCMGCCGCGGTAA** |
| ∟ |  | ◆ | **Patient1** |
| ∟ |  | ◆ | **Patient2** |
| ∟ |  | ◆ | **Patient3** |
| ∟ |  | ◆ | **Patient4** |
| ∟ |  | ◆ | **Patient5** |
| ∟ |  | ◆ | **Patient6** |
| ∟ |  | ◆ | **Patient7** |
| ∟ |  | ◆ | **Patient8** |
| ∟ |  | ◆ | **Patient9** |
| ∟ |  | ◆ | **Patient10** |
| ▶ |  | ■ | **Patient1** |
| ∟ |  | ◆ | **Patient1** |
| ▶ |  | ■ | **Patient2** |
| ∟ |  | ◆ | **Patient2** |
| ▶ |  | ■ | **Patient3** |
| ∟ |  | ◆ | **Patient3** |
| ▶ |  | ■ | **Patient4** |
| ∟ |  | ◆ | **Patient4** |
| ▶ |  | ■ | **Patient5** |
| ∟ |  | ◆ | **Patient5** |
| ▶ |  | ■ | **Patient6** |
| ∟ |  | ◆ | **Patient6** |
| ▶ |  | ■ | **Patient7** |
| ∟ |  | ◆ | **Patient7** |
| ▶ |  | ■ | **Patient8** |
| ∟ |  | ◆ | **Patient8** |
| ▶ |  | ■ | **Patient9** |
| ∟ |  | ◆ | **Patient9** |
| ▶ |  | ■ | **Patient10** |
| ∟ |  | ◆ | **Patient10** |
| ▶ |  | ■ | **NA** |
| ∟ |  | ◆ | **Patient1** |
| ∟ |  | ◆ | **Patient2** |
| ∟ |  | ◆ | **Patient3** |
| ∟ |  | ◆ | **Patient4** |
| ∟ |  | ◆ | **Patient5** |
| ∟ |  | ◆ | **Patient6** |
| ∟ |  | ◆ | **Patient7** |
| ∟ |  | ◆ | **Patient8** |
| ∟ |  | ◆ | **Patient9** |
| ∟ |  | ◆ | **Patient10** |
| ▶ |  | ■ | **GTGCCAGCMGCCGCGGTAA** |
| ∟ |  | ◆ | **Patient1** |
| ∟ |  | ◆ | **Patient2** |
| ∟ |  | ◆ | **Patient3** |
| ∟ |  | ◆ | **Patient4** |
| ∟ |  | ◆ | **Patient5** |
| ∟ |  | ◆ | **Patient6** |
| ∟ |  | ◆ | **Patient7** |
| ∟ |  | ◆ | **Patient8** |
| ∟ |  | ◆ | **Patient9** |
| ∟ |  | ◆ | **Patient10** |
| ▶ |  | ■ | **Patient1** |
| ∟ |  | ◆ | **Patient1** |
| ▶ |  | ■ | **Patient2** |
| ∟ |  | ◆ | **Patient2** |
| ▶ |  | ■ | **Patient3** |
| ∟ |  | ◆ | **Patient3** |
| ▶ |  | ■ | **Patient4** |
| ∟ |  | ◆ | **Patient4** |
| ▶ |  | ■ | **Patient5** |
| ∟ |  | ◆ | **Patient5** |
| ▶ |  | ■ | **Patient6** |
| ∟ |  | ◆ | **Patient6** |
| ▶ |  | ■ | **Patient7** |
| ∟ |  | ◆ | **Patient7** |
| ▶ |  | ■ | **Patient8** |
| ∟ |  | ◆ | **Patient8** |
| ▶ |  | ■ | **Patient9** |
| ∟ |  | ◆ | **Patient9** |
| ▶ |  | ■ | **Patient10** |
| ∟ |  | ◆ | **Patient10** |
| ▶ |  | ■ | **NA** |
| ∟ |  | ◆ | **Patient1** |
| ∟ |  | ◆ | **Patient2** |
| ∟ |  | ◆ | **Patient3** |
| ∟ |  | ◆ | **Patient4** |
| ∟ |  | ◆ | **Patient5** |
| ∟ |  | ◆ | **Patient6** |
| ∟ |  | ◆ | **Patient7** |
| ∟ |  | ◆ | **Patient8** |
| ∟ |  | ◆ | **Patient9** |
| ∟ |  | ◆ | **Patient10** |
| ▶ |  | ■ | **GTGCCAGCMGCCGCGGTAA** |
| ∟ |  | ◆ | **Patient1** |
| ∟ |  | ◆ | **Patient2** |
| ∟ |  | ◆ | **Patient3** |
| ∟ |  | ◆ | **Patient4** |
| ∟ |  | ◆ | **Patient5** |
| ∟ |  | ◆ | **Patient6** |
| ∟ |  | ◆ | **Patient7** |
| ∟ |  | ◆ | **Patient8** |
| ∟ |  | ◆ | **Patient9** |
| ∟ |  | ◆ | **Patient10** |
| ▶ |  | ■ | **Patient1** |
| ∟ |  | ◆ | **Patient1** |
| ▶ |  | ■ | **Patient2** |
| ∟ |  | ◆ | **Patient2** |
| ▶ |  | ■ | **Patient3** |
| ∟ |  | ◆ | **Patient3** |
| ▶ |  | ■ | **Patient4** |
| ∟ |  | ◆ | **Patient4** |
| ▶ |  | ■ | **Patient5** |
| ∟ |  | ◆ | **Patient5** |
| ▶ |  | ■ | **Patient6** |
| ∟ |  | ◆ | **Patient6** |
| ▶ |  | ■ | **Patient7** |
| ∟ |  | ◆ | **Patient7** |
| ▶ |  | ■ | **Patient8** |
| ∟ |  | ◆ | **Patient8** |
| ▶ |  | ■ | **Patient9** |
| ∟ |  | ◆ | **Patient9** |
| ▶ |  | ■ | **Patient10** |
| ∟ |  | ◆ | **Patient10** |
| ▶ |  | ■ | **NA** |
| ∟ |  | ◆ | **Patient1** |
| ∟ |  | ◆ | **Patient2** |
| ∟ |  | ◆ | **Patient3** |
| ∟ |  | ◆ | **Patient4** |
| ∟ |  | ◆ | **Patient5** |
| ∟ |  | ◆ | **Patient6** |
| ∟ |  | ◆ | **Patient7** |
| ∟ |  | ◆ | **Patient8** |
| ∟ |  | ◆ | **Patient9** |
| ∟ |  | ◆ | **Patient10** |
| ▶ |  | ■ | **GTGCCAGCMGCCGCGGTAA** |
| ∟ |  | ◆ | **Patient1** |
| ∟ |  | ◆ | **Patient2** |
| ∟ |  | ◆ | **Patient3** |
| ∟ |  | ◆ | **Patient4** |
| ∟ |  | ◆ | **Patient5** |
| ∟ |  | ◆ | **Patient6** |
| ∟ |  | ◆ | **Patient7** |
| ∟ |  | ◆ | **Patient8** |
| ∟ |  | ◆ | **Patient9** |
| ∟ |  | ◆ | **Patient10** |
| ▶ |  | ■ | **Patient1** |
| ∟ |  | ◆ | **Patient1** |
| ▶ |  | ■ | **Patient2** |
| ∟ |  | ◆ | **Patient2** |
| ▶ |  | ■ | **Patient3** |
| ∟ |  | ◆ | **Patient3** |
| ▶ |  | ■ | **Patient4** |
| ∟ |  | ◆ | **Patient4** |
| ▶ |  | ■ | **Patient5** |
| ∟ |  | ◆ | **Patient5** |
| ▶ |  | ■ | **Patient6** |
| ∟ |  | ◆ | **Patient6** |
| ▶ |  | ■ | **Patient7** |
| ∟ |  | ◆ | **Patient7** |
| ▶ |  | ■ | **Patient8** |
| ∟ |  | ◆ | **Patient8** |
| ▶ |  | ■ | **Patient9** |
| ∟ |  | ◆ | **Patient9** |
| ▶ |  | ■ | **Patient10** |
| ∟ |  | ◆ | **Patient10** |
| ▶ |  | ■ | **NA** |
| ∟ |  | ◆ | **Patient1** |
| ∟ |  | ◆ | **Patient2** |
| ∟ |  | ◆ | **Patient3** |
| ∟ |  | ◆ | **Patient4** |
| ∟ |  | ◆ | **Patient5** |
| ∟ |  | ◆ | **Patient6** |
| ∟ |  | ◆ | **Patient7** |
| ∟ |  | ◆ | **Patient8** |
| ∟ |  | ◆ | **Patient9** |
| ∟ |  | ◆ | **Patient10** |
| ▶ |  | ■ | **GTGCCAGCMGCCGCGGTAA** |
| ∟ |  | ◆ | **Patient1** |
| ∟ |  | ◆ | **Patient2** |
| ∟ |  | ◆ | **Patient3** |
| ∟ |  | ◆ | **Patient4** |
| ∟ |  | ◆ | **Patient5** |
| ∟ |  | ◆ | **Patient6** |
| ∟ |  | ◆ | **Patient7** |
| ∟ |  | ◆ | **Patient8** |
| ∟ |  | ◆ | **Patient9** |
| ∟ |  | ◆ | **Patient10** |
| ▶ |  | ■ | **Patient1** |
| ∟ |  | ◆ | **Patient1** |
| ▶ |  | ■ | **Patient2** |
| ∟ |  | ◆ | **Patient2** |
| ▶ |  | ■ | **Patient3** |
| ∟ |  | ◆ | **Patient3** |
| ▶ |  | ■ | **Patient4** |
| ∟ |  | ◆ | **Patient4** |
| ▶ |  | ■ | **Patient5** |
| ∟ |  | ◆ | **Patient5** |
| ▶ |  | ■ | **Patient6** |
| ∟ |  | ◆ | **Patient6** |
| ▶ |  | ■ | **Patient7** |
| ∟ |  | ◆ | **Patient7** |
| ▶ |  | ■ | **Patient8** |
| ∟ |  | ◆ | **Patient8** |
| ▶ |  | ■ | **Patient9** |
| ∟ |  | ◆ | **Patient9** |
| ▶ |  | ■ | **Patient10** |
| ∟ |  | ◆ | **Patient10** |
| ▶ |  | ■ | **NA** |
| ∟ |  | ◆ | **Patient1** |
| ∟ |  | ◆ | **Patient2** |
| ∟ |  | ◆ | **Patient3** |
| ∟ |  | ◆ | **Patient4** |
| ∟ |  | ◆ | **Patient5** |
| ∟ |  | ◆ | **Patient6** |
| ∟ |  | ◆ | **Patient7** |
| ∟ |  | ◆ | **Patient8** |
| ∟ |  | ◆ | **Patient9** |
| ∟ |  | ◆ | **Patient10** |
| ▶ |  | ■ | **GTGCCAGCMGCCGCGGTAA** |
| ∟ |  | ◆ | **Patient1** |
| ∟ |  | ◆ | **Patient2** |
| ∟ |  | ◆ | **Patient3** |
| ∟ |  | ◆ | **Patient4** |
| ∟ |  | ◆ | **Patient5** |
| ∟ |  | ◆ | **Patient6** |
| ∟ |  | ◆ | **Patient7** |
| ∟ |  | ◆ | **Patient8** |
| ∟ |  | ◆ | **Patient9** |
| ∟ |  | ◆ | **Patient10** |
| ▶ |  | ■ | **Patient1** |
| ∟ |  | ◆ | **Patient1** |
| ▶ |  | ■ | **Patient2** |
| ∟ |  | ◆ | **Patient2** |
| ▶ |  | ■ | **Patient3** |
| ∟ |  | ◆ | **Patient3** |
| ▶ |  | ■ | **Patient4** |
| ∟ |  | ◆ | **Patient4** |
| ▶ |  | ■ | **Patient5** |
| ∟ |  | ◆ | **Patient5** |
| ▶ |  | ■ | **Patient6** |
| ∟ |  | ◆ | **Patient6** |
| ▶ |  | ■ | **Patient7** |
| ∟ |  | ◆ | **Patient7** |
| ▶ |  | ■ | **Patient8** |
| ∟ |  | ◆ | **Patient8** |
| ▶ |  | ■ | **Patient9** |
| ∟ |  | ◆ | **Patient9** |
| ▶ |  | ■ | **Patient10** |
| ∟ |  | ◆ | **Patient10** |

**If the lines for some categories do not extend all the way to the right end of the x-axis, that means that at least one of the samples in that category does not have that many sequences.**

  
  

|  |  |  |  |  |  |  |  |  |  |  |  |  |  |  |  |  |  |
| --- | --- | --- | --- | --- | --- | --- | --- | --- | --- | --- | --- | --- | --- | --- | --- | --- | --- |
| LinkerPrimerSequence | Seqs/Sample | PD\_whole\_tree Ave. | PD\_whole\_tree Err. | chao1 Ave. | chao1 Err. | fisher\_alpha Ave. | fisher\_alpha Err. | observed\_species Ave. | observed\_species Err. | shannon Ave. | shannon Err. | simpson Ave. | simpson Err. | simpson\_e Ave. | simpson\_e Err. | simpson\_reciprocal Ave. | simpson\_reciprocal Err. |
| GTGCCAGCMGCCGCGGTAA | 10.0 | 0.491 | 0.239 | 7.677 | 2.314 | 5.907 | 2.783 | 5.270 | 1.034 | 2.114 | 0.371 | 0.718 | 0.083 | 0.770 | 0.047 | 4.095 | 1.042 || GTGCCAGCMGCCGCGGTAA | 2009.0 | 1.331 | 0.260 | 21.824 | 4.553 | 3.213 | 0.817 | 20.580 | 4.438 | 2.936 | 0.604 | 0.797 | 0.094 | 0.291 | 0.100 | 6.071 | 2.627 || GTGCCAGCMGCCGCGGTAA | 4008.0 | 1.443 | 0.281 | 22.486 | 4.454 | 3.076 | 0.722 | 21.980 | 4.430 | 2.941 | 0.600 | 0.798 | 0.093 | 0.274 | 0.100 | 6.067 | 2.612 || GTGCCAGCMGCCGCGGTAA | 6007.0 | 1.494 | 0.327 | 22.783 | 4.491 | 2.979 | 0.682 | 22.590 | 4.488 | 2.942 | 0.601 | 0.798 | 0.093 | 0.267 | 0.099 | 6.075 | 2.608 || GTGCCAGCMGCCGCGGTAA | 8006.0 | 1.496 | 0.314 | 22.682 | 4.461 | 2.858 | 0.652 | 22.610 | 4.501 | 2.943 | 0.603 | 0.798 | 0.093 | 0.268 | 0.101 | 6.090 | 2.631 || GTGCCAGCMGCCGCGGTAA | 10005.0 | 1.511 | 0.328 | 22.768 | 4.435 | 2.783 | 0.625 | 22.720 | 4.467 | 2.945 | 0.603 | 0.798 | 0.093 | 0.267 | 0.101 | 6.091 | 2.631 || GTGCCAGCMGCCGCGGTAA | 12004.0 | 1.519 | 0.333 | 22.790 | 4.465 | 2.724 | 0.607 | 22.790 | 4.465 | 2.945 | 0.602 | 0.798 | 0.093 | 0.266 | 0.100 | 6.087 | 2.612 || GTGCCAGCMGCCGCGGTAA | 14003.0 | 1.520 | 0.333 | 22.800 | 4.468 | 2.669 | 0.594 | 22.800 | 4.468 | 2.944 | 0.601 | 0.798 | 0.093 | 0.265 | 0.100 | 6.078 | 2.617 || GTGCCAGCMGCCGCGGTAA | 16002.0 | 1.520 | 0.333 | 22.790 | 4.467 | 2.622 | 0.582 | 22.790 | 4.467 | 2.944 | 0.601 | 0.798 | 0.093 | 0.266 | 0.101 | 6.086 | 2.623 || GTGCCAGCMGCCGCGGTAA | 18001.0 | 1.520 | 0.333 | 22.800 | 4.468 | 2.584 | 0.572 | 22.800 | 4.468 | 2.947 | 0.601 | 0.799 | 0.093 | 0.266 | 0.101 | 6.095 | 2.627 || GTGCCAGCMGCCGCGGTAA | 20000.0 | 1.520 | 0.333 | 22.800 | 4.468 | 2.549 | 0.564 | 22.800 | 4.468 | 2.946 | 0.601 | 0.798 | 0.093 | 0.266 | 0.101 | 6.089 | 2.627 |
| BarcodeSequence | Seqs/Sample | PD\_whole\_tree Ave. | PD\_whole\_tree Err. | chao1 Ave. | chao1 Err. | fisher\_alpha Ave. | fisher\_alpha Err. | observed\_species Ave. | observed\_species Err. | shannon Ave. | shannon Err. | simpson Ave. | simpson Err. | simpson\_e Ave. | simpson\_e Err. | simpson\_reciprocal Ave. | simpson\_reciprocal Err. |
| NA | 10.0 | 0.491 | 0.239 | 7.677 | 2.314 | 5.907 | 2.783 | 5.270 | 1.034 | 2.114 | 0.371 | 0.718 | 0.083 | 0.770 | 0.047 | 4.095 | 1.042 || NA | 2009.0 | 1.331 | 0.260 | 21.824 | 4.553 | 3.213 | 0.817 | 20.580 | 4.438 | 2.936 | 0.604 | 0.797 | 0.094 | 0.291 | 0.100 | 6.071 | 2.627 || NA | 4008.0 | 1.443 | 0.281 | 22.486 | 4.454 | 3.076 | 0.722 | 21.980 | 4.430 | 2.941 | 0.600 | 0.798 | 0.093 | 0.274 | 0.100 | 6.067 | 2.612 || NA | 6007.0 | 1.494 | 0.327 | 22.783 | 4.491 | 2.979 | 0.682 | 22.590 | 4.488 | 2.942 | 0.601 | 0.798 | 0.093 | 0.267 | 0.099 | 6.075 | 2.608 || NA | 8006.0 | 1.496 | 0.314 | 22.682 | 4.461 | 2.858 | 0.652 | 22.610 | 4.501 | 2.943 | 0.603 | 0.798 | 0.093 | 0.268 | 0.101 | 6.090 | 2.631 || NA | 10005.0 | 1.511 | 0.328 | 22.768 | 4.435 | 2.783 | 0.625 | 22.720 | 4.467 | 2.945 | 0.603 | 0.798 | 0.093 | 0.267 | 0.101 | 6.091 | 2.631 || NA | 12004.0 | 1.519 | 0.333 | 22.790 | 4.465 | 2.724 | 0.607 | 22.790 | 4.465 | 2.945 | 0.602 | 0.798 | 0.093 | 0.266 | 0.100 | 6.087 | 2.612 || NA | 14003.0 | 1.520 | 0.333 | 22.800 | 4.468 | 2.669 | 0.594 | 22.800 | 4.468 | 2.944 | 0.601 | 0.798 | 0.093 | 0.265 | 0.100 | 6.078 | 2.617 || NA | 16002.0 | 1.520 | 0.333 | 22.790 | 4.467 | 2.622 | 0.582 | 22.790 | 4.467 | 2.944 | 0.601 | 0.798 | 0.093 | 0.266 | 0.101 | 6.086 | 2.623 || NA | 18001.0 | 1.520 | 0.333 | 22.800 | 4.468 | 2.584 | 0.572 | 22.800 | 4.468 | 2.947 | 0.601 | 0.799 | 0.093 | 0.266 | 0.101 | 6.095 | 2.627 || NA | 20000.0 | 1.520 | 0.333 | 22.800 | 4.468 | 2.549 | 0.564 | 22.800 | 4.468 | 2.946 | 0.601 | 0.798 | 0.093 | 0.266 | 0.101 | 6.089 | 2.627 |
| SampleID | Seqs/Sample | PD\_whole\_tree Ave. | PD\_whole\_tree Err. | chao1 Ave. | chao1 Err. | fisher\_alpha Ave. | fisher\_alpha Err. | observed\_species Ave. | observed\_species Err. | shannon Ave. | shannon Err. | simpson Ave. | simpson Err. | simpson\_e Ave. | simpson\_e Err. | simpson\_reciprocal Ave. | simpson\_reciprocal Err. |
| Patient1 | 10.0 | 0.254 | nan | 6.600 | nan | 3.594 | nan | 4.400 | nan | 1.788 | nan | 0.648 | nan | 0.703 | nan | 3.039 | nan || Patient1 | 2009.0 | 1.791 | nan | 31.162 | nan | 4.745 | nan | 28.700 | nan | 2.919 | nan | 0.770 | nan | 0.152 | nan | 4.350 | nan || Patient1 | 4008.0 | 2.032 | nan | 31.300 | nan | 4.557 | nan | 30.900 | nan | 2.939 | nan | 0.773 | nan | 0.143 | nan | 4.407 | nan || Patient1 | 6007.0 | 2.224 | nan | 31.675 | nan | 4.373 | nan | 31.600 | nan | 2.929 | nan | 0.771 | nan | 0.139 | nan | 4.376 | nan || Patient1 | 8006.0 | 2.179 | nan | 31.833 | nan | 4.196 | nan | 31.700 | nan | 2.925 | nan | 0.771 | nan | 0.138 | nan | 4.360 | nan || Patient1 | 10005.0 | 2.245 | nan | 31.900 | nan | 4.088 | nan | 31.900 | nan | 2.937 | nan | 0.772 | nan | 0.137 | nan | 4.378 | nan || Patient1 | 12004.0 | 2.266 | nan | 32.000 | nan | 3.996 | nan | 32.000 | nan | 2.939 | nan | 0.772 | nan | 0.137 | nan | 4.396 | nan || Patient1 | 14003.0 | 2.266 | nan | 32.000 | nan | 3.910 | nan | 32.000 | nan | 2.930 | nan | 0.771 | nan | 0.136 | nan | 4.367 | nan || Patient1 | 16002.0 | 2.266 | nan | 32.000 | nan | 3.839 | nan | 32.000 | nan | 2.926 | nan | 0.771 | nan | 0.136 | nan | 4.365 | nan || Patient1 | 18001.0 | 2.266 | nan | 32.000 | nan | 3.778 | nan | 32.000 | nan | 2.932 | nan | 0.772 | nan | 0.137 | nan | 4.378 | nan || Patient1 | 20000.0 | 2.266 | nan | 32.000 | nan | 3.726 | nan | 32.000 | nan | 2.932 | nan | 0.771 | nan | 0.137 | nan | 4.372 | nan || Patient2 | 10.0 | 0.571 | nan | 7.033 | nan | 5.404 | nan | 5.100 | nan | 2.072 | nan | 0.714 | nan | 0.790 | nan | 3.991 | nan || Patient2 | 2009.0 | 1.114 | nan | 18.333 | nan | 2.762 | nan | 18.200 | nan | 2.929 | nan | 0.812 | nan | 0.293 | nan | 5.334 | nan || Patient2 | 4008.0 | 1.122 | nan | 19.000 | nan | 2.555 | nan | 18.800 | nan | 2.910 | nan | 0.809 | nan | 0.279 | nan | 5.246 | nan || Patient2 | 6007.0 | 1.123 | nan | 18.900 | nan | 2.417 | nan | 18.900 | nan | 2.929 | nan | 0.812 | nan | 0.281 | nan | 5.306 | nan || Patient2 | 8006.0 | 1.123 | nan | 18.900 | nan | 2.320 | nan | 18.900 | nan | 2.924 | nan | 0.811 | nan | 0.280 | nan | 5.288 | nan || Patient2 | 10005.0 | 1.125 | nan | 19.000 | nan | 2.263 | nan | 19.000 | nan | 2.932 | nan | 0.812 | nan | 0.279 | nan | 5.310 | nan || Patient2 | 12004.0 | 1.125 | nan | 19.000 | nan | 2.209 | nan | 19.000 | nan | 2.930 | nan | 0.812 | nan | 0.280 | nan | 5.316 | nan || Patient2 | 14003.0 | 1.125 | nan | 19.000 | nan | 2.165 | nan | 19.000 | nan | 2.923 | nan | 0.811 | nan | 0.278 | nan | 5.286 | nan || Patient2 | 16002.0 | 1.125 | nan | 19.000 | nan | 2.129 | nan | 19.000 | nan | 2.926 | nan | 0.811 | nan | 0.279 | nan | 5.296 | nan || Patient2 | 18001.0 | 1.125 | nan | 19.000 | nan | 2.098 | nan | 19.000 | nan | 2.924 | nan | 0.811 | nan | 0.278 | nan | 5.288 | nan || Patient2 | 20000.0 | 1.125 | nan | 19.000 | nan | 2.071 | nan | 19.000 | nan | 2.923 | nan | 0.811 | nan | 0.278 | nan | 5.279 | nan || Patient3 | 10.0 | 0.713 | nan | 8.067 | nan | 7.407 | nan | 6.100 | nan | 2.437 | nan | 0.792 | nan | 0.819 | nan | 5.029 | nan || Patient3 | 2009.0 | 1.415 | nan | 22.183 | nan | 3.289 | nan | 21.100 | nan | 3.512 | nan | 0.892 | nan | 0.438 | nan | 9.237 | nan || Patient3 | 4008.0 | 1.437 | nan | 22.033 | nan | 3.033 | nan | 21.800 | nan | 3.516 | nan | 0.892 | nan | 0.423 | nan | 9.225 | nan || Patient3 | 6007.0 | 1.442 | nan | 22.000 | nan | 2.878 | nan | 22.000 | nan | 3.518 | nan | 0.892 | nan | 0.423 | nan | 9.301 | nan || Patient3 | 8006.0 | 1.442 | nan | 22.000 | nan | 2.759 | nan | 22.000 | nan | 3.520 | nan | 0.892 | nan | 0.422 | nan | 9.294 | nan || Patient3 | 10005.0 | 1.442 | nan | 22.000 | nan | 2.674 | nan | 22.000 | nan | 3.525 | nan | 0.893 | nan | 0.424 | nan | 9.318 | nan || Patient3 | 12004.0 | 1.442 | nan | 22.000 | nan | 2.608 | nan | 22.000 | nan | 3.518 | nan | 0.892 | nan | 0.422 | nan | 9.274 | nan || Patient3 | 14003.0 | 1.442 | nan | 22.000 | nan | 2.555 | nan | 22.000 | nan | 3.518 | nan | 0.892 | nan | 0.420 | nan | 9.247 | nan || Patient3 | 16002.0 | 1.442 | nan | 22.000 | nan | 2.511 | nan | 22.000 | nan | 3.520 | nan | 0.892 | nan | 0.422 | nan | 9.292 | nan || Patient3 | 18001.0 | 1.442 | nan | 22.000 | nan | 2.474 | nan | 22.000 | nan | 3.524 | nan | 0.893 | nan | 0.423 | nan | 9.303 | nan || Patient3 | 20000.0 | 1.442 | nan | 22.000 | nan | 2.441 | nan | 22.000 | nan | 3.525 | nan | 0.893 | nan | 0.424 | nan | 9.322 | nan || Patient4 | 10.0 | 0.836 | nan | 11.333 | nan | 8.965 | nan | 6.300 | nan | 2.471 | nan | 0.796 | nan | 0.811 | nan | 5.101 | nan || Patient4 | 2009.0 | 1.633 | nan | 23.808 | nan | 3.531 | nan | 22.400 | nan | 3.520 | nan | 0.889 | nan | 0.402 | nan | 9.000 | nan || Patient4 | 4008.0 | 1.683 | nan | 23.475 | nan | 3.261 | nan | 23.200 | nan | 3.520 | nan | 0.889 | nan | 0.388 | nan | 8.987 | nan || Patient4 | 6007.0 | 1.739 | nan | 24.100 | nan | 3.181 | nan | 24.000 | nan | 3.515 | nan | 0.888 | nan | 0.373 | nan | 8.945 | nan || Patient4 | 8006.0 | 1.732 | nan | 23.900 | nan | 3.034 | nan | 23.900 | nan | 3.522 | nan | 0.889 | nan | 0.378 | nan | 9.029 | nan || Patient4 | 10005.0 | 1.727 | nan | 23.800 | nan | 2.925 | nan | 23.800 | nan | 3.517 | nan | 0.889 | nan | 0.377 | nan | 8.980 | nan || Patient4 | 12004.0 | 1.739 | nan | 24.000 | nan | 2.879 | nan | 24.000 | nan | 3.516 | nan | 0.888 | nan | 0.373 | nan | 8.949 | nan || Patient4 | 14003.0 | 1.739 | nan | 24.000 | nan | 2.820 | nan | 24.000 | nan | 3.514 | nan | 0.888 | nan | 0.373 | nan | 8.951 | nan || Patient4 | 16002.0 | 1.739 | nan | 24.000 | nan | 2.771 | nan | 24.000 | nan | 3.516 | nan | 0.888 | nan | 0.373 | nan | 8.961 | nan || Patient4 | 18001.0 | 1.739 | nan | 24.000 | nan | 2.729 | nan | 24.000 | nan | 3.520 | nan | 0.889 | nan | 0.374 | nan | 8.981 | nan || Patient4 | 20000.0 | 1.739 | nan | 24.000 | nan | 2.693 | nan | 24.000 | nan | 3.518 | nan | 0.889 | nan | 0.374 | nan | 8.971 | nan || Patient5 | 10.0 | 0.737 | nan | 10.100 | nan | 10.157 | nan | 6.700 | nan | 2.577 | nan | 0.810 | nan | 0.812 | nan | 5.470 | nan || Patient5 | 2009.0 | 1.279 | nan | 21.633 | nan | 3.253 | nan | 20.900 | nan | 3.489 | nan | 0.881 | nan | 0.403 | nan | 8.412 | nan || Patient5 | 4008.0 | 1.336 | nan | 23.683 | nan | 3.098 | nan | 22.200 | nan | 3.479 | nan | 0.880 | nan | 0.377 | nan | 8.355 | nan || Patient5 | 6007.0 | 1.343 | nan | 23.033 | nan | 2.984 | nan | 22.700 | nan | 3.481 | nan | 0.880 | nan | 0.368 | nan | 8.342 | nan || Patient5 | 8006.0 | 1.333 | nan | 22.750 | nan | 2.860 | nan | 22.700 | nan | 3.488 | nan | 0.881 | nan | 0.370 | nan | 8.400 | nan || Patient5 | 10005.0 | 1.343 | nan | 23.000 | nan | 2.799 | nan | 22.900 | nan | 3.486 | nan | 0.881 | nan | 0.367 | nan | 8.403 | nan || Patient5 | 12004.0 | 1.343 | nan | 23.000 | nan | 2.743 | nan | 23.000 | nan | 3.491 | nan | 0.881 | nan | 0.366 | nan | 8.425 | nan || Patient5 | 14003.0 | 1.343 | nan | 23.000 | nan | 2.687 | nan | 23.000 | nan | 3.490 | nan | 0.881 | nan | 0.366 | nan | 8.415 | nan || Patient5 | 16002.0 | 1.343 | nan | 22.900 | nan | 2.628 | nan | 22.900 | nan | 3.489 | nan | 0.881 | nan | 0.368 | nan | 8.423 | nan || Patient5 | 18001.0 | 1.343 | nan | 23.000 | nan | 2.601 | nan | 23.000 | nan | 3.493 | nan | 0.882 | nan | 0.367 | nan | 8.450 | nan || Patient5 | 20000.0 | 1.343 | nan | 23.000 | nan | 2.567 | nan | 23.000 | nan | 3.489 | nan | 0.881 | nan | 0.366 | nan | 8.417 | nan || Patient6 | 10.0 | 0.161 | nan | 3.900 | nan | 1.973 | nan | 3.400 | nan | 1.390 | nan | 0.544 | nan | 0.703 | nan | 2.363 | nan || Patient6 | 2009.0 | 0.991 | nan | 14.500 | nan | 1.812 | nan | 12.700 | nan | 1.710 | nan | 0.599 | nan | 0.197 | nan | 2.498 | nan || Patient6 | 4008.0 | 1.224 | nan | 15.183 | nan | 1.909 | nan | 14.600 | nan | 1.720 | nan | 0.600 | nan | 0.172 | nan | 2.500 | nan || Patient6 | 6007.0 | 1.282 | nan | 15.625 | nan | 1.913 | nan | 15.400 | nan | 1.703 | nan | 0.598 | nan | 0.162 | nan | 2.489 | nan || Patient6 | 8006.0 | 1.356 | nan | 15.833 | nan | 1.838 | nan | 15.400 | nan | 1.708 | nan | 0.599 | nan | 0.163 | nan | 2.496 | nan || Patient6 | 10005.0 | 1.390 | nan | 16.000 | nan | 1.837 | nan | 15.800 | nan | 1.710 | nan | 0.599 | nan | 0.158 | nan | 2.494 | nan || Patient6 | 12004.0 | 1.421 | nan | 16.000 | nan | 1.819 | nan | 16.000 | nan | 1.705 | nan | 0.597 | nan | 0.155 | nan | 2.482 | nan || Patient6 | 14003.0 | 1.421 | nan | 16.000 | nan | 1.784 | nan | 16.000 | nan | 1.708 | nan | 0.598 | nan | 0.155 | nan | 2.487 | nan || Patient6 | 16002.0 | 1.421 | nan | 16.000 | nan | 1.755 | nan | 16.000 | nan | 1.713 | nan | 0.600 | nan | 0.156 | nan | 2.499 | nan || Patient6 | 18001.0 | 1.421 | nan | 16.000 | nan | 1.730 | nan | 16.000 | nan | 1.719 | nan | 0.601 | nan | 0.157 | nan | 2.505 | nan || Patient6 | 20000.0 | 1.421 | nan | 16.000 | nan | 1.708 | nan | 16.000 | nan | 1.717 | nan | 0.600 | nan | 0.156 | nan | 2.501 | nan || Patient7 | 10.0 | 0.314 | nan | 6.133 | nan | 3.910 | nan | 4.700 | nan | 1.930 | nan | 0.682 | nan | 0.745 | nan | 3.462 | nan || Patient7 | 2009.0 | 1.391 | nan | 22.237 | nan | 3.218 | nan | 20.700 | nan | 2.680 | nan | 0.758 | nan | 0.201 | nan | 4.142 | nan || Patient7 | 4008.0 | 1.521 | nan | 22.942 | nan | 3.115 | nan | 22.300 | nan | 2.698 | nan | 0.761 | nan | 0.188 | nan | 4.183 | nan || Patient7 | 6007.0 | 1.599 | nan | 23.983 | nan | 3.136 | nan | 23.700 | nan | 2.704 | nan | 0.762 | nan | 0.178 | nan | 4.209 | nan || Patient7 | 8006.0 | 1.625 | nan | 24.050 | nan | 3.048 | nan | 24.000 | nan | 2.701 | nan | 0.761 | nan | 0.175 | nan | 4.193 | nan || Patient7 | 10005.0 | 1.605 | nan | 23.833 | nan | 2.925 | nan | 23.800 | nan | 2.700 | nan | 0.761 | nan | 0.176 | nan | 4.181 | nan || Patient7 | 12004.0 | 1.622 | nan | 23.900 | nan | 2.866 | nan | 23.900 | nan | 2.706 | nan | 0.763 | nan | 0.176 | nan | 4.215 | nan || Patient7 | 14003.0 | 1.625 | nan | 24.000 | nan | 2.820 | nan | 24.000 | nan | 2.706 | nan | 0.762 | nan | 0.175 | nan | 4.198 | nan || Patient7 | 16002.0 | 1.625 | nan | 24.000 | nan | 2.771 | nan | 24.000 | nan | 2.705 | nan | 0.762 | nan | 0.175 | nan | 4.193 | nan || Patient7 | 18001.0 | 1.625 | nan | 24.000 | nan | 2.729 | nan | 24.000 | nan | 2.712 | nan | 0.763 | nan | 0.176 | nan | 4.212 | nan || Patient7 | 20000.0 | 1.625 | nan | 24.000 | nan | 2.693 | nan | 24.000 | nan | 2.708 | nan | 0.762 | nan | 0.175 | nan | 4.206 | nan || Patient8 | 10.0 | 0.292 | nan | 6.270 | nan | 4.721 | nan | 5.100 | nan | 2.133 | nan | 0.738 | nan | 0.792 | nan | 4.059 | nan || Patient8 | 2009.0 | 1.336 | nan | 20.333 | nan | 2.729 | nan | 18.000 | nan | 2.642 | nan | 0.781 | nan | 0.256 | nan | 4.568 | nan || Patient8 | 4008.0 | 1.573 | nan | 21.295 | nan | 2.825 | nan | 20.500 | nan | 2.652 | nan | 0.783 | nan | 0.225 | nan | 4.601 | nan || Patient8 | 6007.0 | 1.660 | nan | 22.310 | nan | 2.818 | nan | 21.600 | nan | 2.662 | nan | 0.784 | nan | 0.214 | nan | 4.621 | nan || Patient8 | 8006.0 | 1.657 | nan | 21.650 | nan | 2.702 | nan | 21.600 | nan | 2.652 | nan | 0.783 | nan | 0.213 | nan | 4.606 | nan || Patient8 | 10005.0 | 1.702 | nan | 22.150 | nan | 2.674 | nan | 22.000 | nan | 2.664 | nan | 0.784 | nan | 0.210 | nan | 4.625 | nan || Patient8 | 12004.0 | 1.702 | nan | 22.000 | nan | 2.608 | nan | 22.000 | nan | 2.664 | nan | 0.784 | nan | 0.211 | nan | 4.635 | nan || Patient8 | 14003.0 | 1.702 | nan | 22.000 | nan | 2.555 | nan | 22.000 | nan | 2.662 | nan | 0.784 | nan | 0.210 | nan | 4.620 | nan || Patient8 | 16002.0 | 1.702 | nan | 22.000 | nan | 2.511 | nan | 22.000 | nan | 2.663 | nan | 0.784 | nan | 0.210 | nan | 4.621 | nan || Patient8 | 18001.0 | 1.702 | nan | 22.000 | nan | 2.474 | nan | 22.000 | nan | 2.661 | nan | 0.784 | nan | 0.210 | nan | 4.620 | nan || Patient8 | 20000.0 | 1.702 | nan | 22.000 | nan | 2.441 | nan | 22.000 | nan | 2.659 | nan | 0.783 | nan | 0.210 | nan | 4.612 | nan || Patient9 | 10.0 | 0.740 | nan | 11.117 | nan | 9.686 | nan | 6.500 | nan | 2.529 | nan | 0.804 | nan | 0.818 | nan | 5.341 | nan || Patient9 | 2009.0 | 1.457 | nan | 26.908 | nan | 4.332 | nan | 26.600 | nan | 3.690 | nan | 0.899 | nan | 0.373 | nan | 9.914 | nan || Patient9 | 4008.0 | 1.500 | nan | 28.000 | nan | 3.993 | nan | 27.600 | nan | 3.696 | nan | 0.899 | nan | 0.359 | nan | 9.908 | nan || Patient9 | 6007.0 | 1.520 | nan | 28.200 | nan | 3.801 | nan | 28.000 | nan | 3.692 | nan | 0.899 | nan | 0.353 | nan | 9.882 | nan || Patient9 | 8006.0 | 1.502 | nan | 27.900 | nan | 3.623 | nan | 27.900 | nan | 3.700 | nan | 0.899 | nan | 0.356 | nan | 9.939 | nan || Patient9 | 10005.0 | 1.520 | nan | 28.000 | nan | 3.521 | nan | 28.000 | nan | 3.701 | nan | 0.899 | nan | 0.355 | nan | 9.950 | nan || Patient9 | 12004.0 | 1.520 | nan | 28.000 | nan | 3.431 | nan | 28.000 | nan | 3.695 | nan | 0.899 | nan | 0.353 | nan | 9.894 | nan || Patient9 | 14003.0 | 1.520 | nan | 28.000 | nan | 3.359 | nan | 28.000 | nan | 3.698 | nan | 0.899 | nan | 0.354 | nan | 9.925 | nan || Patient9 | 16002.0 | 1.520 | nan | 28.000 | nan | 3.299 | nan | 28.000 | nan | 3.698 | nan | 0.899 | nan | 0.354 | nan | 9.925 | nan || Patient9 | 18001.0 | 1.520 | nan | 28.000 | nan | 3.248 | nan | 28.000 | nan | 3.700 | nan | 0.899 | nan | 0.355 | nan | 9.935 | nan || Patient9 | 20000.0 | 1.520 | nan | 28.000 | nan | 3.204 | nan | 28.000 | nan | 3.700 | nan | 0.899 | nan | 0.355 | nan | 9.932 | nan || Patient10 | 10.0 | 0.297 | nan | 6.217 | nan | 3.257 | nan | 4.400 | nan | 1.813 | nan | 0.656 | nan | 0.707 | nan | 3.093 | nan || Patient10 | 2009.0 | 0.907 | nan | 17.142 | nan | 2.461 | nan | 16.500 | nan | 2.271 | nan | 0.693 | nan | 0.198 | nan | 3.260 | nan || Patient10 | 4008.0 | 0.997 | nan | 17.950 | nan | 2.414 | nan | 17.900 | nan | 2.278 | nan | 0.693 | nan | 0.182 | nan | 3.262 | nan || Patient10 | 6007.0 | 1.013 | nan | 18.000 | nan | 2.286 | nan | 18.000 | nan | 2.284 | nan | 0.695 | nan | 0.182 | nan | 3.279 | nan || Patient10 | 8006.0 | 1.013 | nan | 18.000 | nan | 2.195 | nan | 18.000 | nan | 2.290 | nan | 0.697 | nan | 0.183 | nan | 3.296 | nan || Patient10 | 10005.0 | 1.013 | nan | 18.000 | nan | 2.129 | nan | 18.000 | nan | 2.279 | nan | 0.694 | nan | 0.182 | nan | 3.272 | nan || Patient10 | 12004.0 | 1.013 | nan | 18.000 | nan | 2.078 | nan | 18.000 | nan | 2.286 | nan | 0.695 | nan | 0.182 | nan | 3.283 | nan || Patient10 | 14003.0 | 1.013 | nan | 18.000 | nan | 2.037 | nan | 18.000 | nan | 2.287 | nan | 0.696 | nan | 0.182 | nan | 3.285 | nan || Patient10 | 16002.0 | 1.013 | nan | 18.000 | nan | 2.003 | nan | 18.000 | nan | 2.285 | nan | 0.695 | nan | 0.182 | nan | 3.283 | nan || Patient10 | 18001.0 | 1.013 | nan | 18.000 | nan | 1.974 | nan | 18.000 | nan | 2.284 | nan | 0.695 | nan | 0.182 | nan | 3.282 | nan || Patient10 | 20000.0 | 1.013 | nan | 18.000 | nan | 1.949 | nan | 18.000 | nan | 2.285 | nan | 0.695 | nan | 0.182 | nan | 3.282 | nan |
