# Supplementary figures and images for: Baseline Characterization of the Gut Microbiota of Field and Colony Populations of Phlebotomus tobbi and Preliminary Assessment of the Anti-Leishmanial Activity of Cultivable Bacteria
Source: Pathogens. 2026 Jun 23;15(7):658. doi: 10.3390/pathogens15070658 (PMC13416292; doi:10.3390/pathogens15070658)

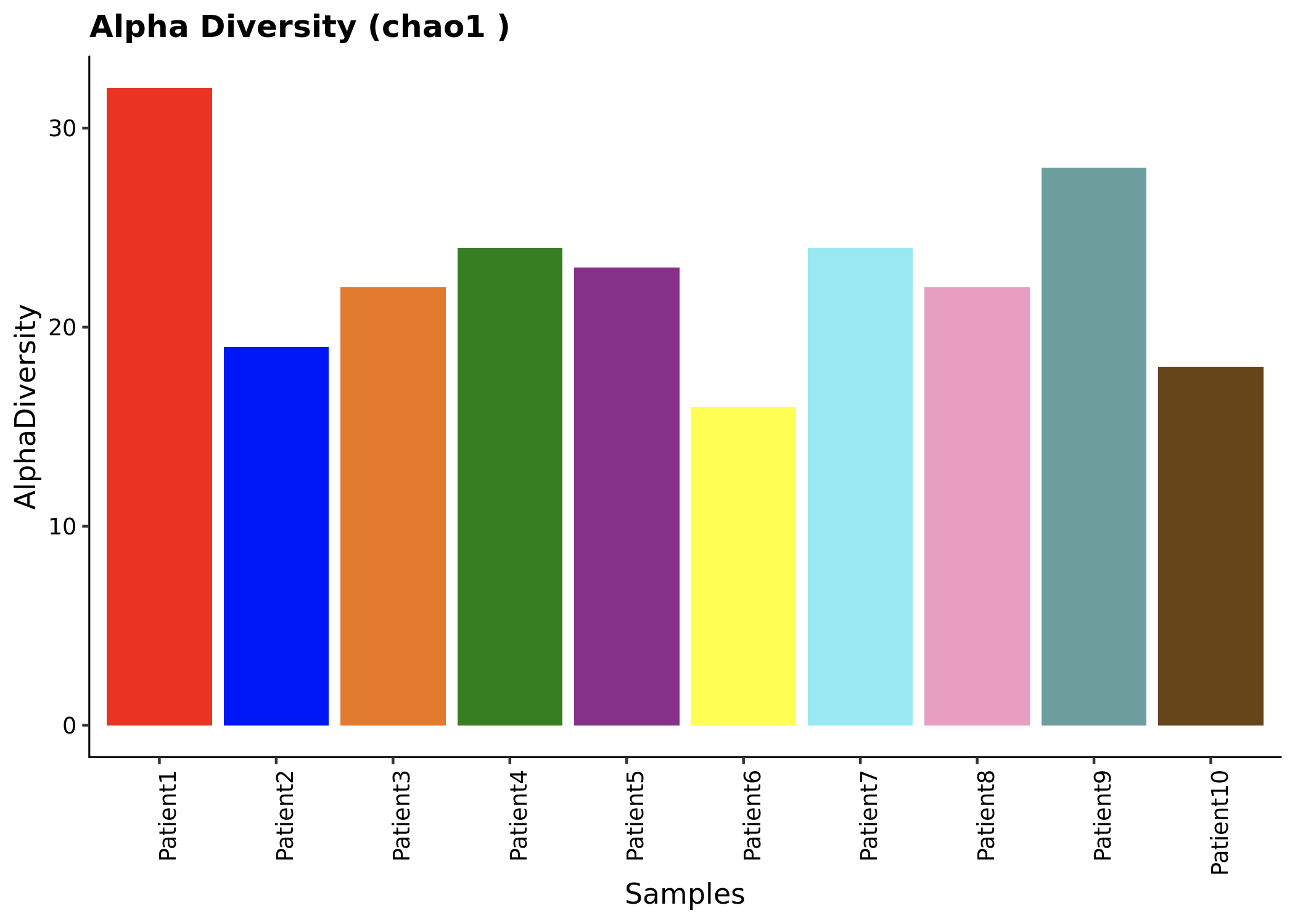

Supplement: Supplementary file 1 [file pathogens-15-00658-s001.zip › pathogens-4357168-File S1/Alpha_Diversity/Boxplots/chao1.png]

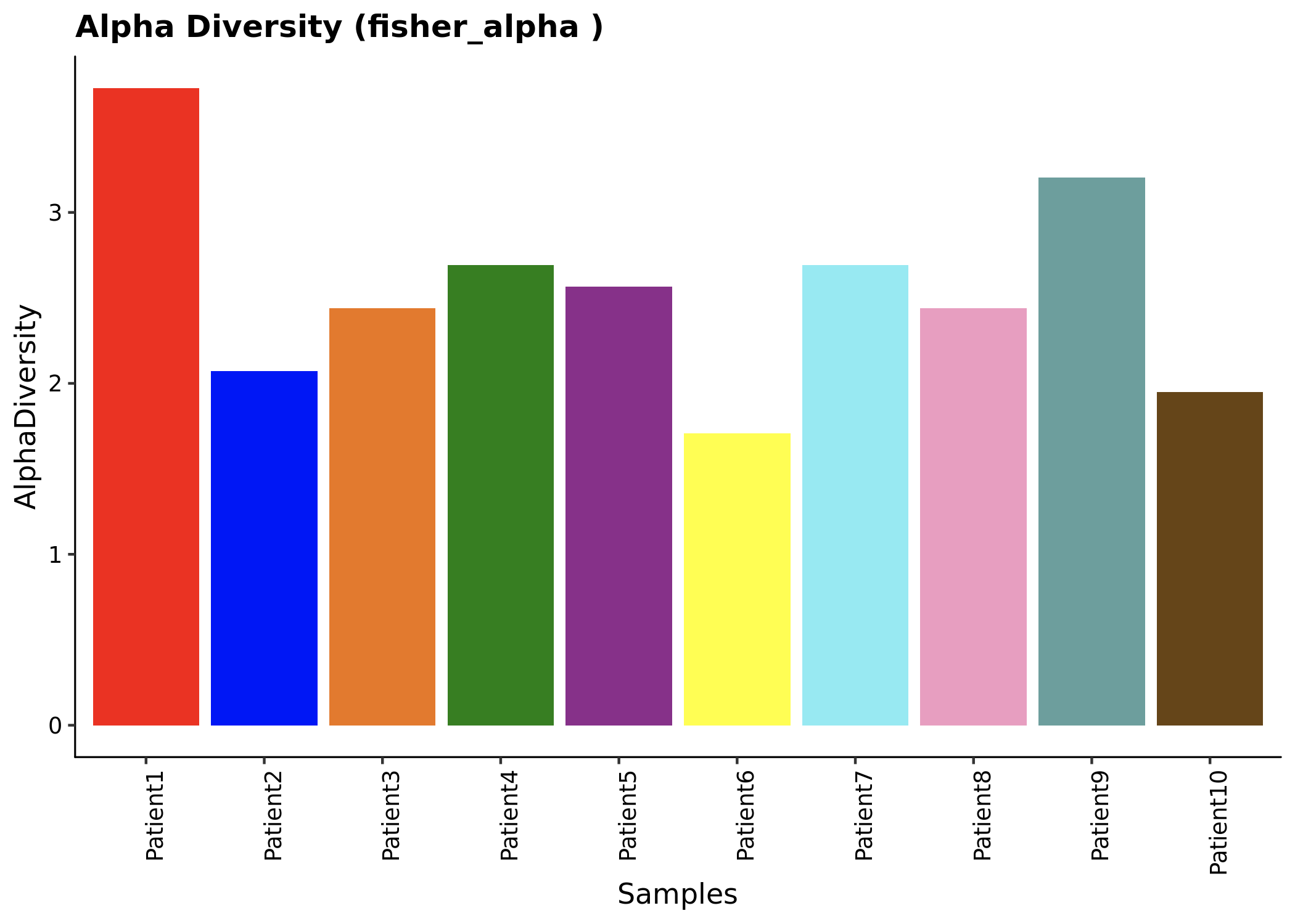

Supplement: Supplementary file 1 [file pathogens-15-00658-s001.zip › pathogens-4357168-File S1/Alpha_Diversity/Boxplots/fisher_alpha.png]

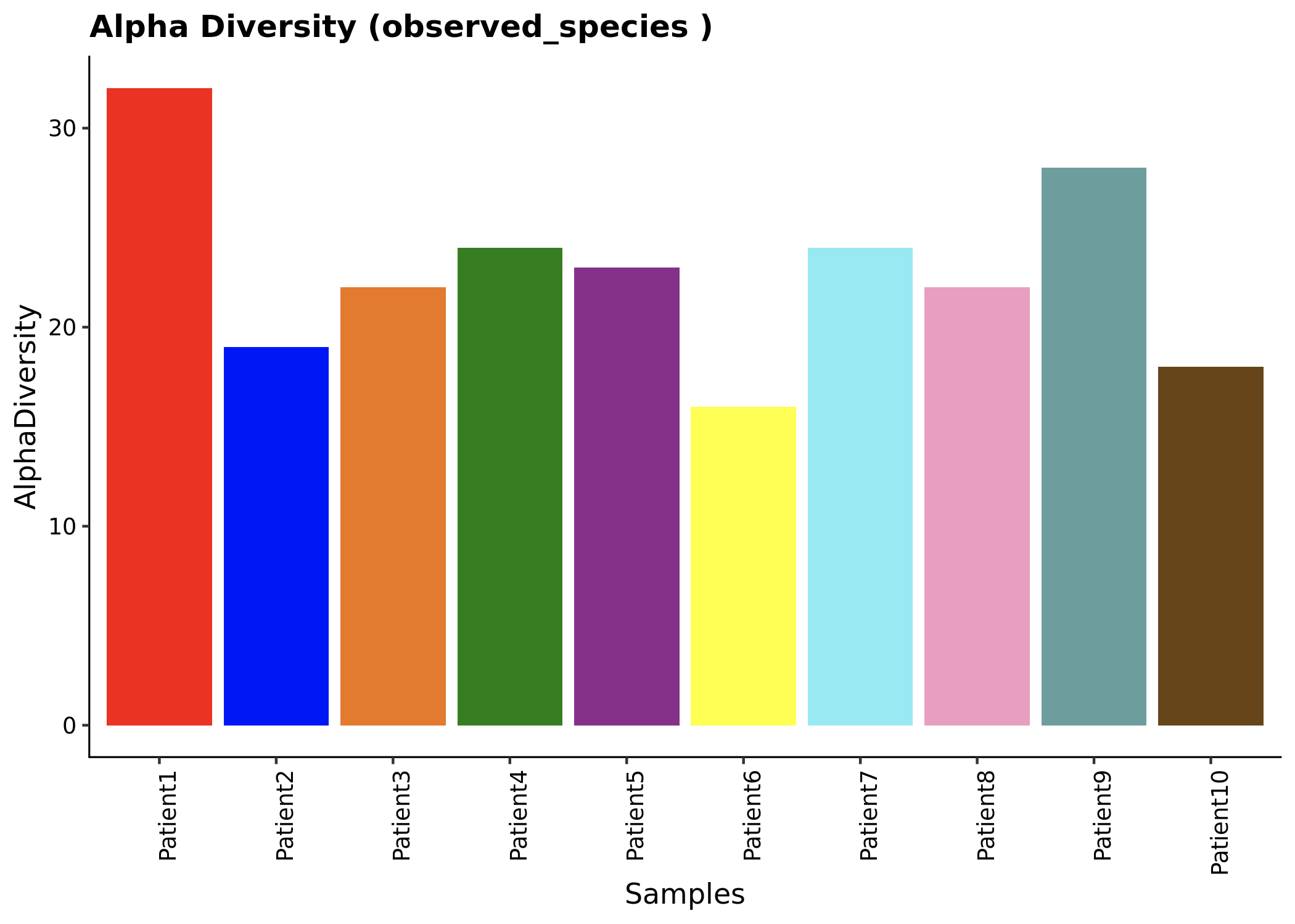

Supplement: Supplementary file 1 [file pathogens-15-00658-s001.zip › pathogens-4357168-File S1/Alpha_Diversity/Boxplots/observed_species.png]

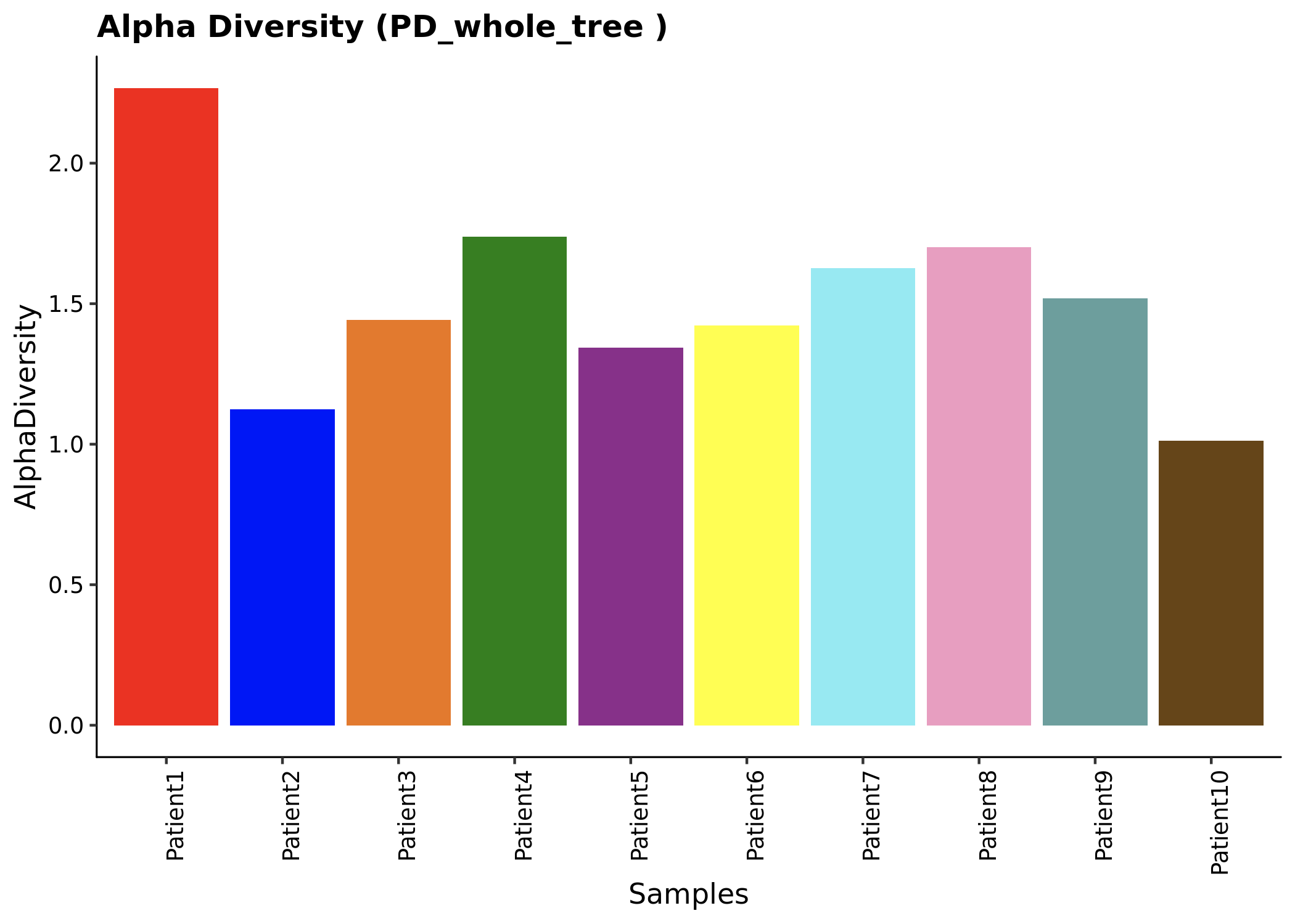

Supplement: Supplementary file 1 [file pathogens-15-00658-s001.zip › pathogens-4357168-File S1/Alpha_Diversity/Boxplots/PD_whole_tree.png]

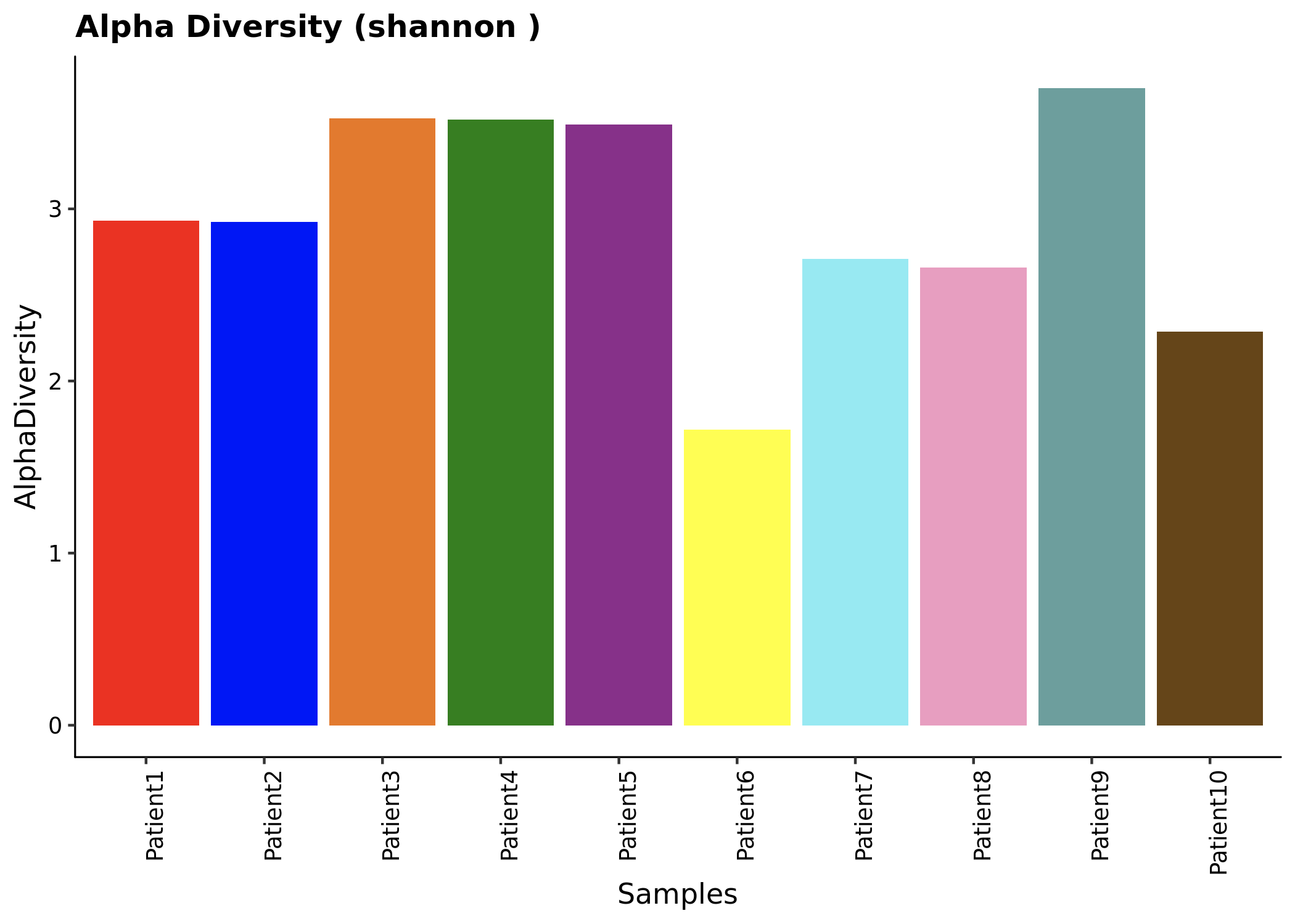

Supplement: Supplementary file 1 [file pathogens-15-00658-s001.zip › pathogens-4357168-File S1/Alpha_Diversity/Boxplots/shannon.png]

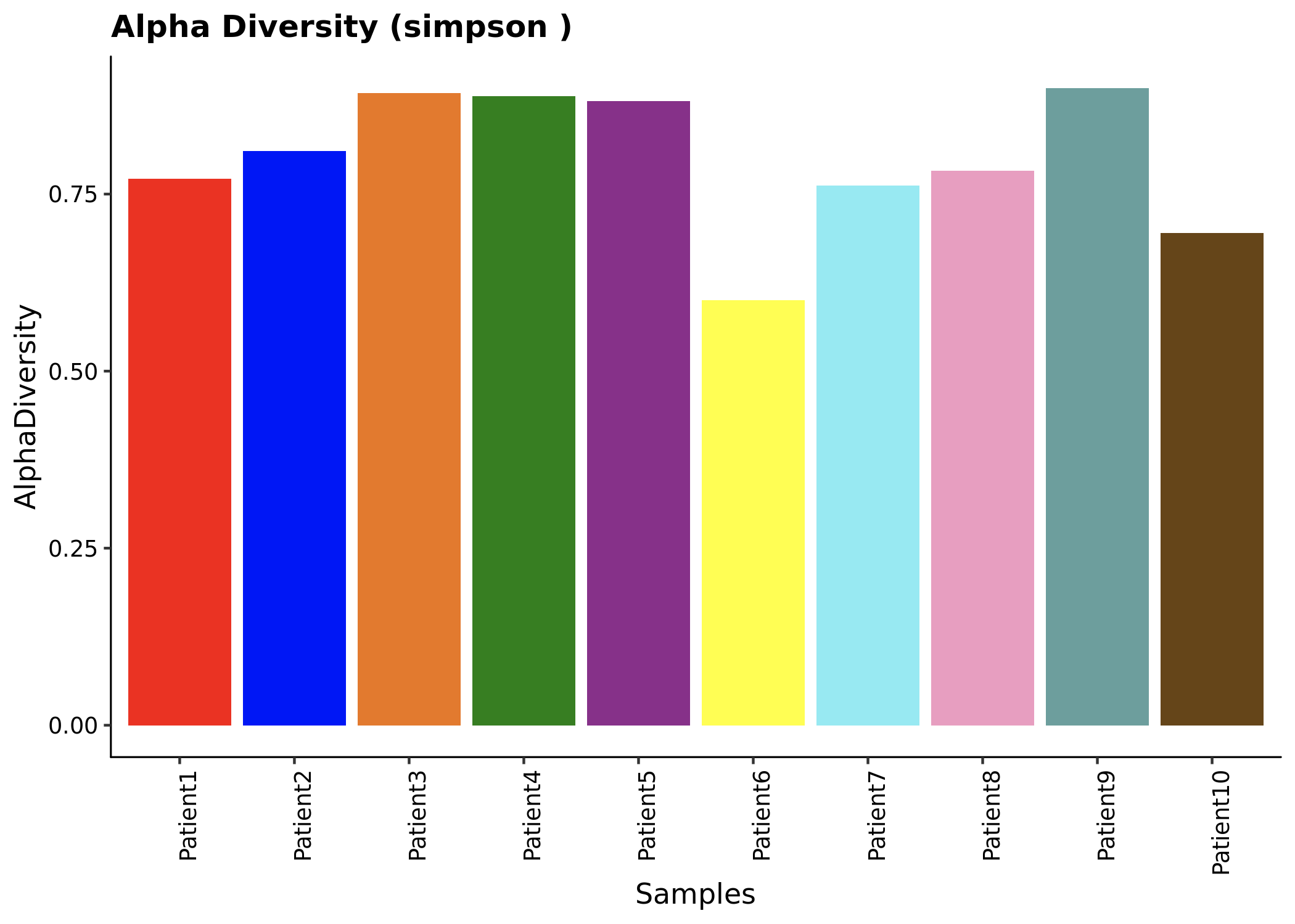

Supplement: Supplementary file 1 [file pathogens-15-00658-s001.zip › pathogens-4357168-File S1/Alpha_Diversity/Boxplots/simpson.png]

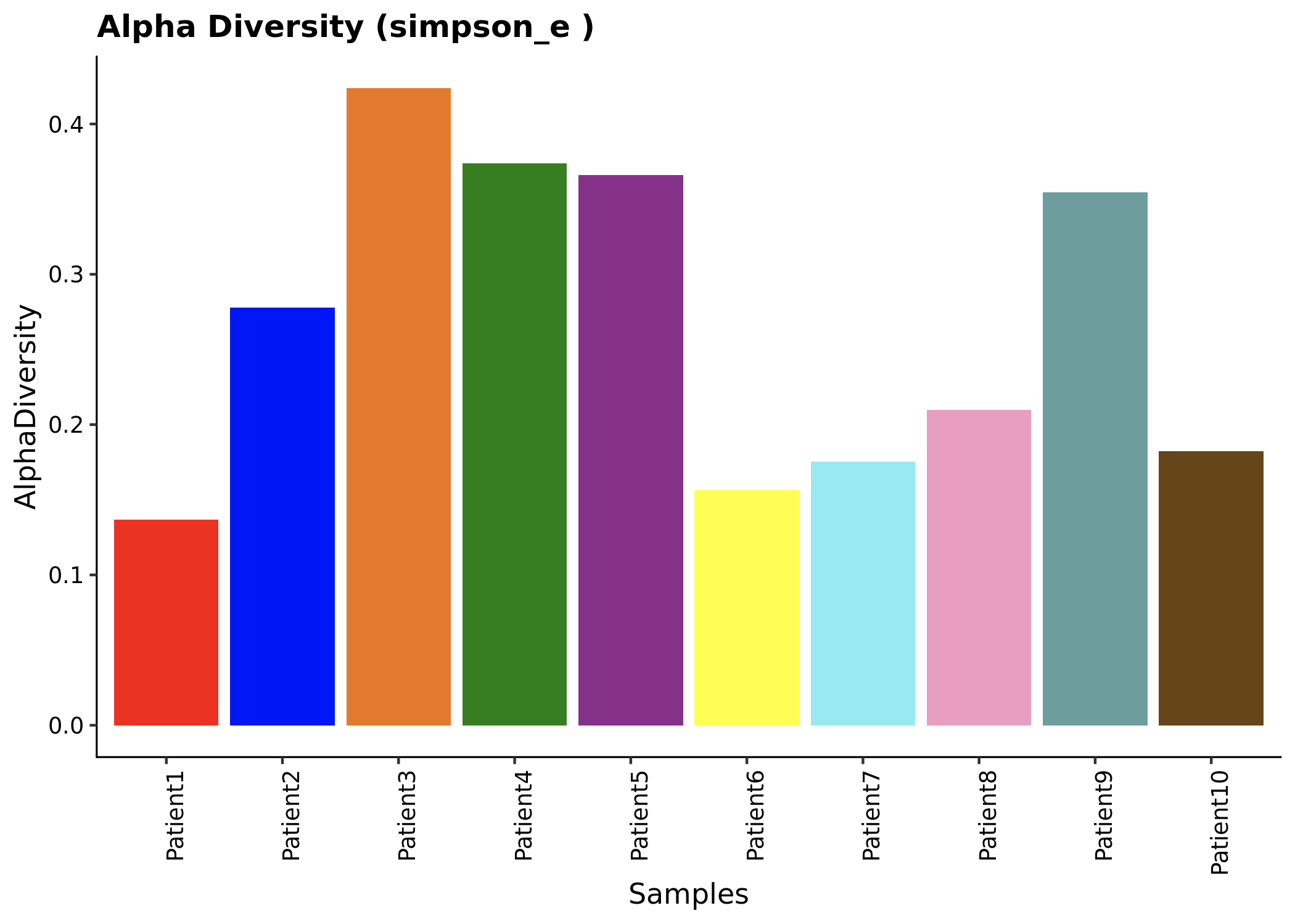

Supplement: Supplementary file 1 [file pathogens-15-00658-s001.zip › pathogens-4357168-File S1/Alpha_Diversity/Boxplots/simpson_e.png]

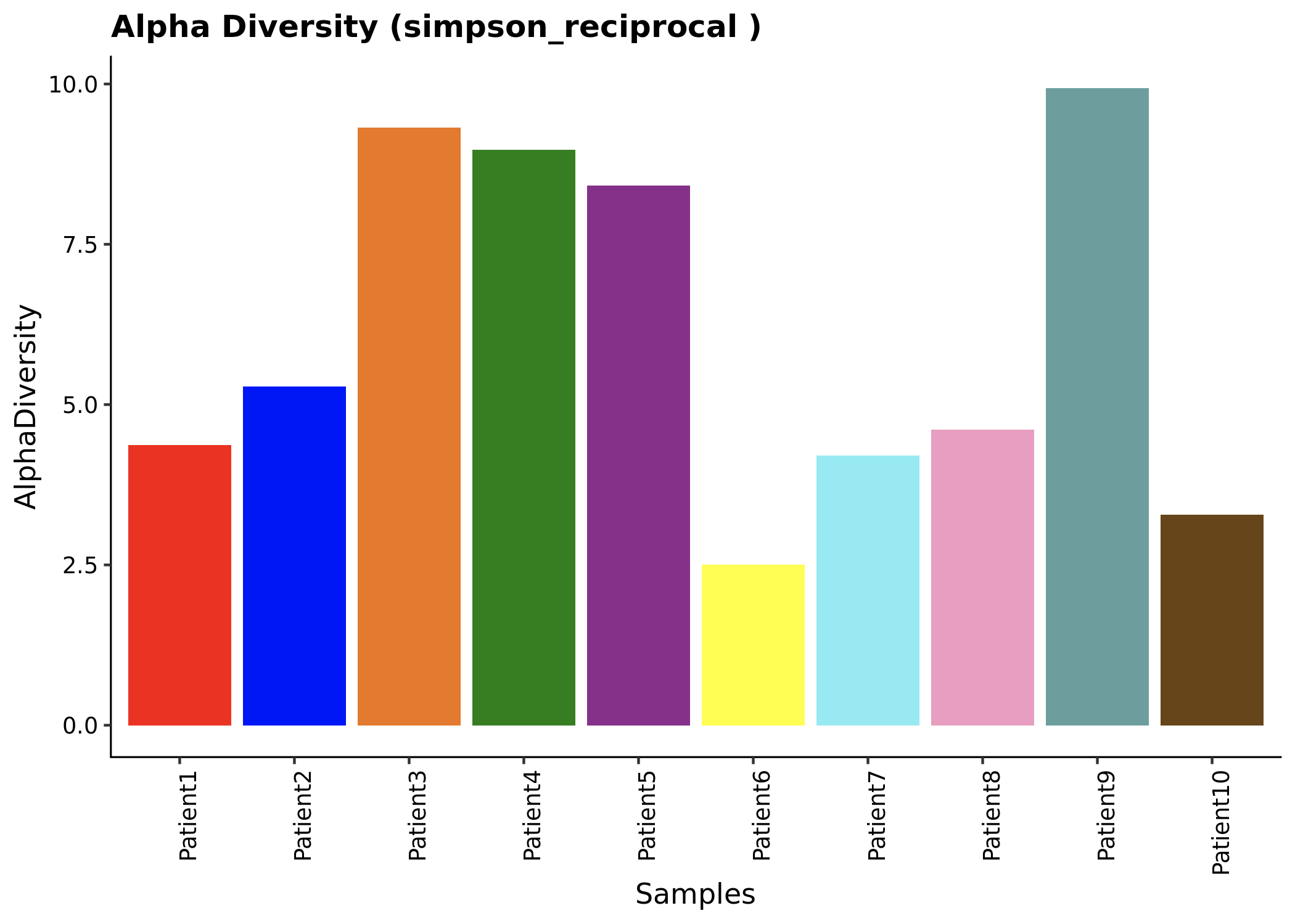

Supplement: Supplementary file 1 [file pathogens-15-00658-s001.zip › pathogens-4357168-File S1/Alpha_Diversity/Boxplots/simpson_reciprocal.png]

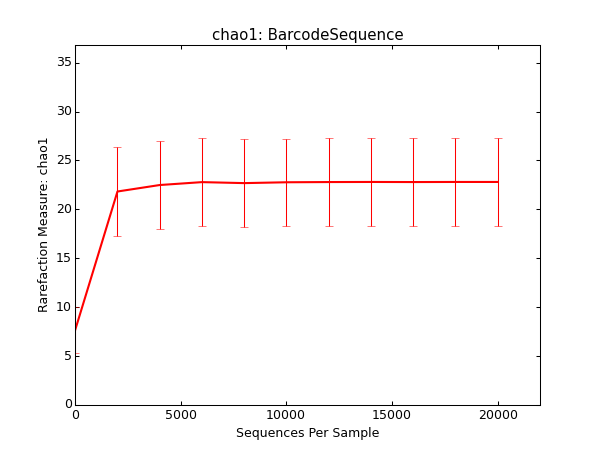

Supplement: Supplementary file 1 [file pathogens-15-00658-s001.zip › pathogens-4357168-File S1/Alpha_Diversity/Rarefaction_Plots/average_plots/chao1BarcodeSequence.png]

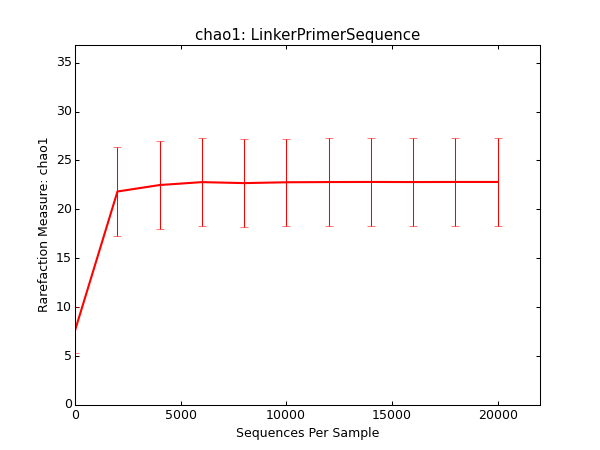

Supplement: Supplementary file 1 [file pathogens-15-00658-s001.zip › pathogens-4357168-File S1/Alpha_Diversity/Rarefaction_Plots/average_plots/chao1LinkerPrimerSequence.png]

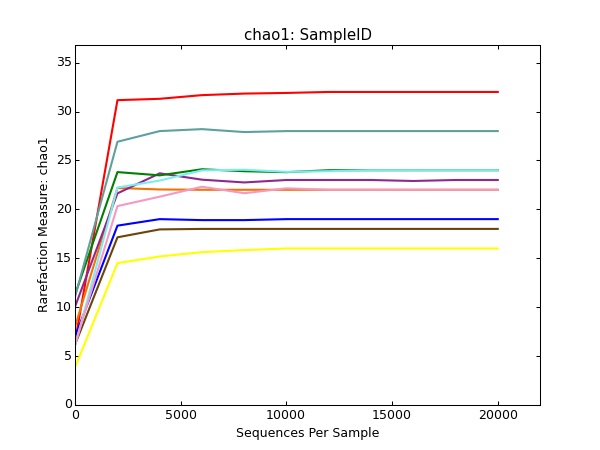

Supplement: Supplementary file 1 [file pathogens-15-00658-s001.zip › pathogens-4357168-File S1/Alpha_Diversity/Rarefaction_Plots/average_plots/chao1SampleID.png]

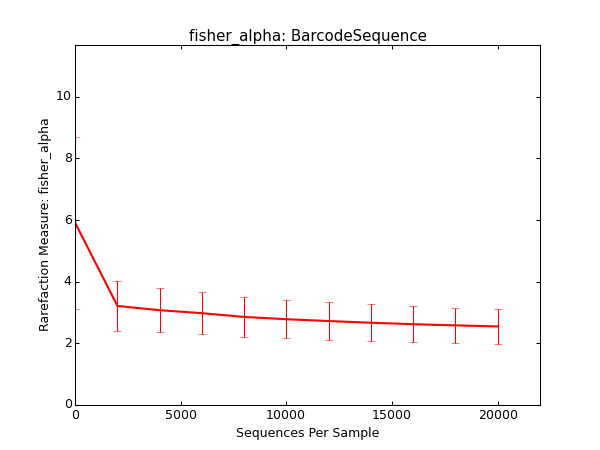

Supplement: Supplementary file 1 [file pathogens-15-00658-s001.zip › pathogens-4357168-File S1/Alpha_Diversity/Rarefaction_Plots/average_plots/fisher_alphaBarcodeSequence.png]

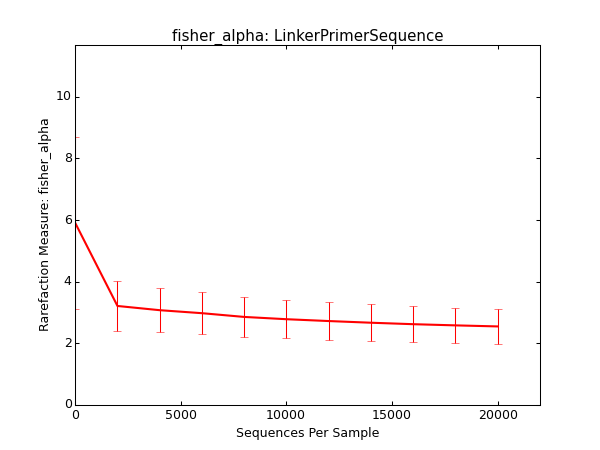

Supplement: Supplementary file 1 [file pathogens-15-00658-s001.zip › pathogens-4357168-File S1/Alpha_Diversity/Rarefaction_Plots/average_plots/fisher_alphaLinkerPrimerSequence.png]

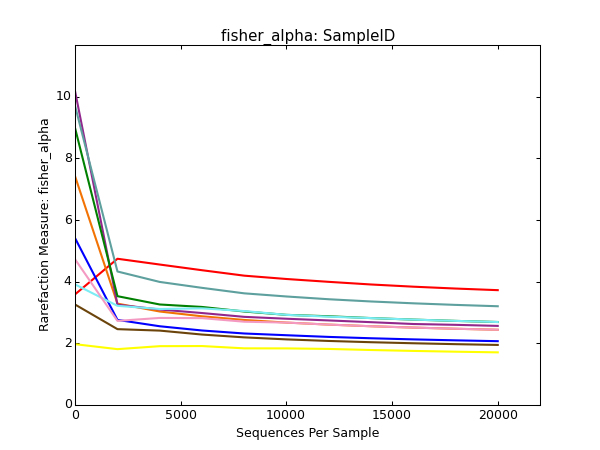

Supplement: Supplementary file 1 [file pathogens-15-00658-s001.zip › pathogens-4357168-File S1/Alpha_Diversity/Rarefaction_Plots/average_plots/fisher_alphaSampleID.png]

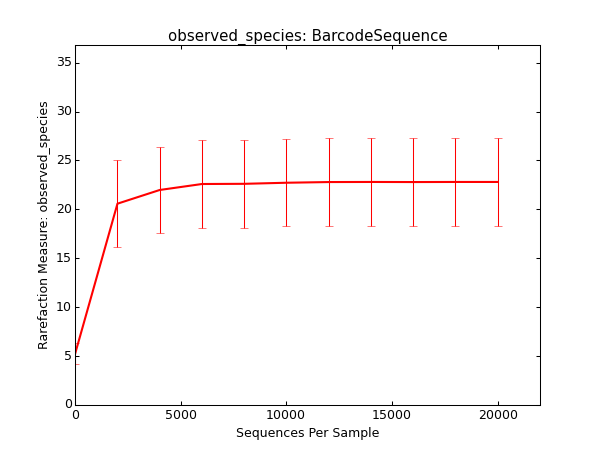

Supplement: Supplementary file 1 [file pathogens-15-00658-s001.zip › pathogens-4357168-File S1/Alpha_Diversity/Rarefaction_Plots/average_plots/observed_speciesBarcodeSequence.png]

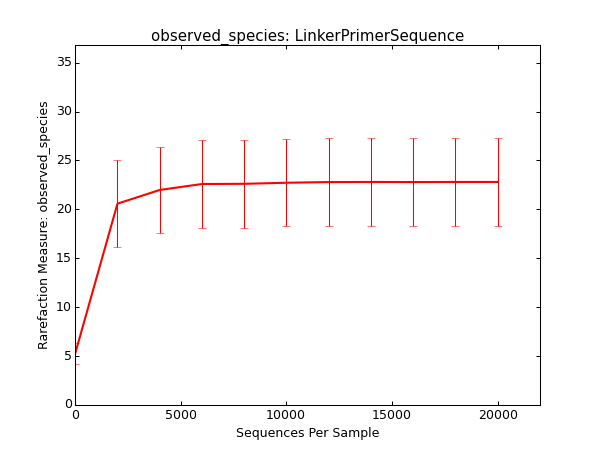

Supplement: Supplementary file 1 [file pathogens-15-00658-s001.zip › pathogens-4357168-File S1/Alpha_Diversity/Rarefaction_Plots/average_plots/observed_speciesLinkerPrimerSequence.png]

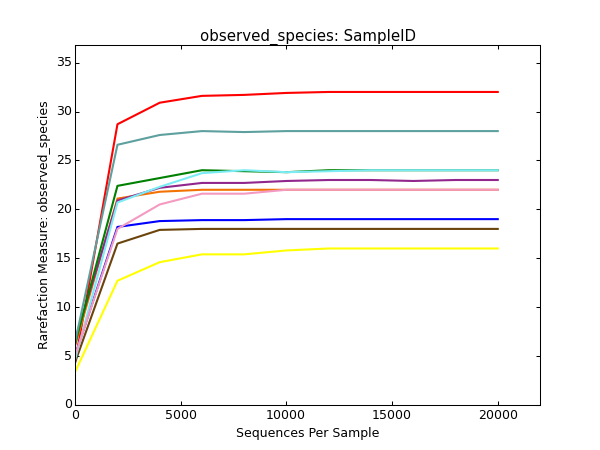

Supplement: Supplementary file 1 [file pathogens-15-00658-s001.zip › pathogens-4357168-File S1/Alpha_Diversity/Rarefaction_Plots/average_plots/observed_speciesSampleID.png]

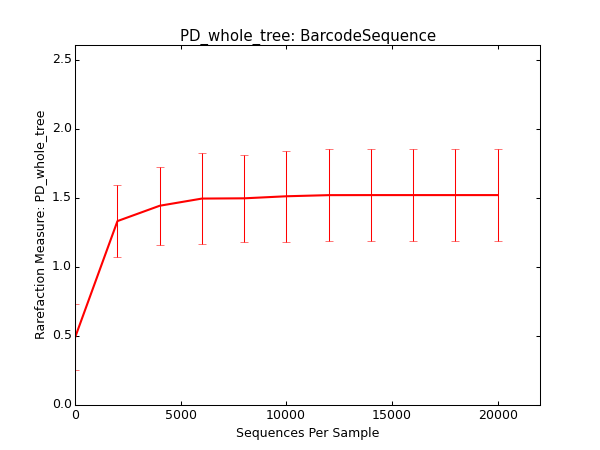

Supplement: Supplementary file 1 [file pathogens-15-00658-s001.zip › pathogens-4357168-File S1/Alpha_Diversity/Rarefaction_Plots/average_plots/PD_whole_treeBarcodeSequence.png]

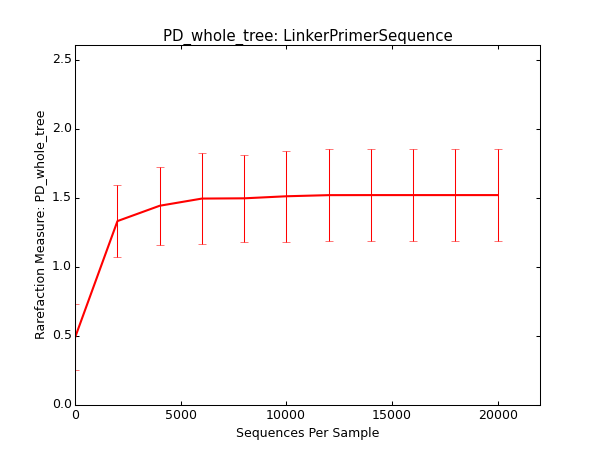

Supplement: Supplementary file 1 [file pathogens-15-00658-s001.zip › pathogens-4357168-File S1/Alpha_Diversity/Rarefaction_Plots/average_plots/PD_whole_treeLinkerPrimerSequence.png]

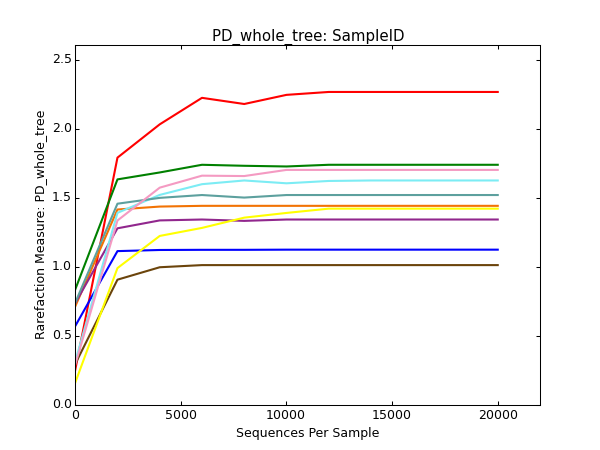

Supplement: Supplementary file 1 [file pathogens-15-00658-s001.zip › pathogens-4357168-File S1/Alpha_Diversity/Rarefaction_Plots/average_plots/PD_whole_treeSampleID.png]

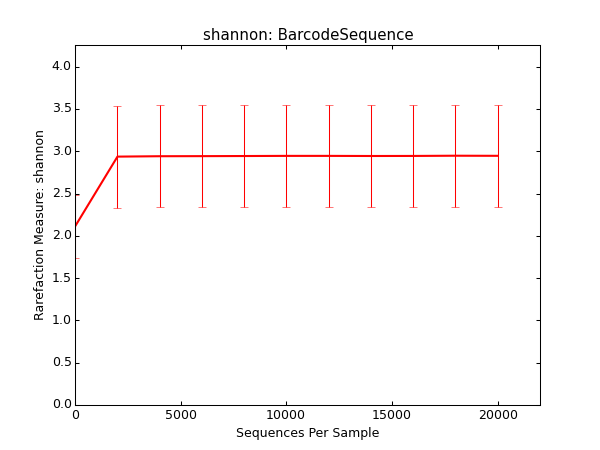

Supplement: Supplementary file 1 [file pathogens-15-00658-s001.zip › pathogens-4357168-File S1/Alpha_Diversity/Rarefaction_Plots/average_plots/shannonBarcodeSequence.png]

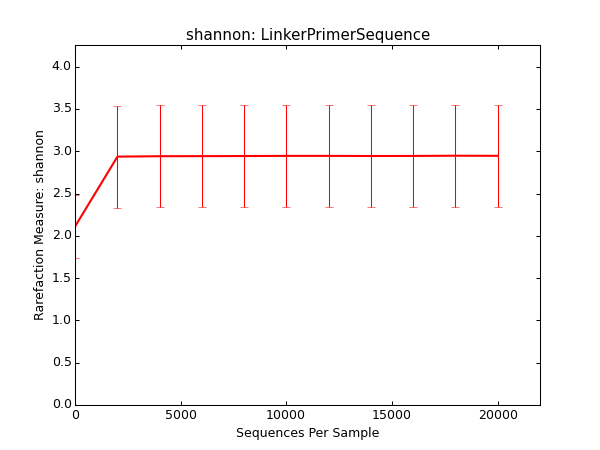

Supplement: Supplementary file 1 [file pathogens-15-00658-s001.zip › pathogens-4357168-File S1/Alpha_Diversity/Rarefaction_Plots/average_plots/shannonLinkerPrimerSequence.png]

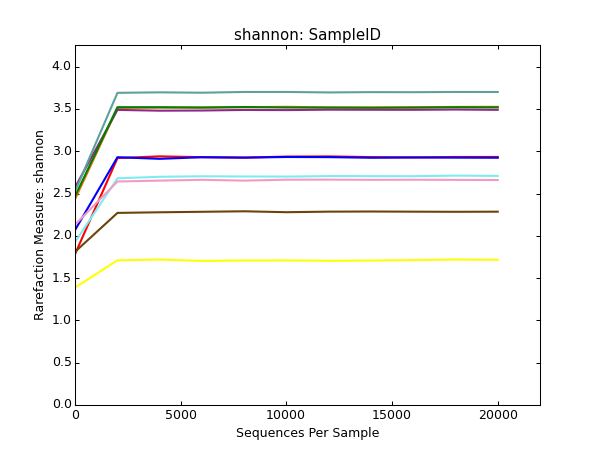

Supplement: Supplementary file 1 [file pathogens-15-00658-s001.zip › pathogens-4357168-File S1/Alpha_Diversity/Rarefaction_Plots/average_plots/shannonSampleID.png]

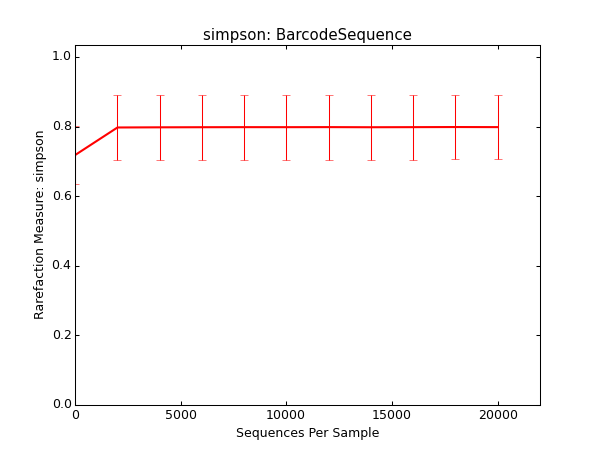

Supplement: Supplementary file 1 [file pathogens-15-00658-s001.zip › pathogens-4357168-File S1/Alpha_Diversity/Rarefaction_Plots/average_plots/simpsonBarcodeSequence.png]

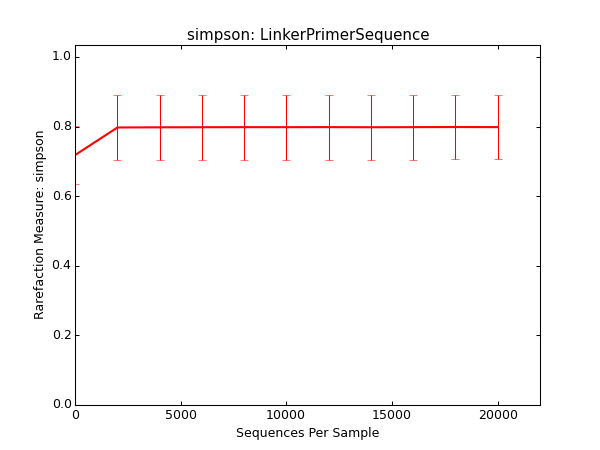

Supplement: Supplementary file 1 [file pathogens-15-00658-s001.zip › pathogens-4357168-File S1/Alpha_Diversity/Rarefaction_Plots/average_plots/simpsonLinkerPrimerSequence.png]

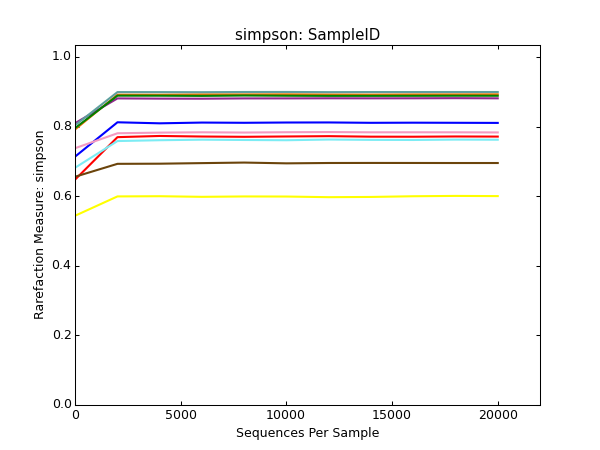

Supplement: Supplementary file 1 [file pathogens-15-00658-s001.zip › pathogens-4357168-File S1/Alpha_Diversity/Rarefaction_Plots/average_plots/simpsonSampleID.png]

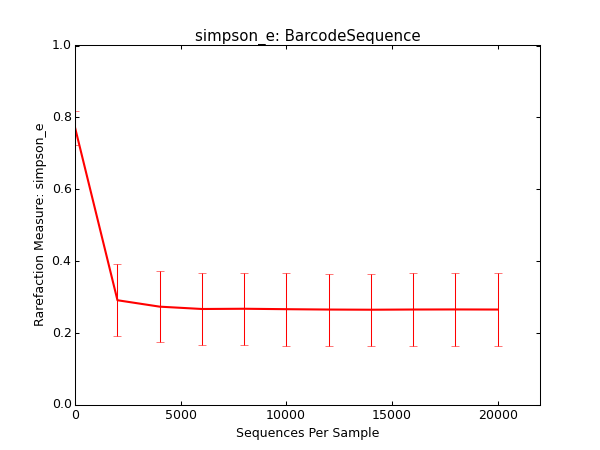

Supplement: Supplementary file 1 [file pathogens-15-00658-s001.zip › pathogens-4357168-File S1/Alpha_Diversity/Rarefaction_Plots/average_plots/simpson_eBarcodeSequence.png]

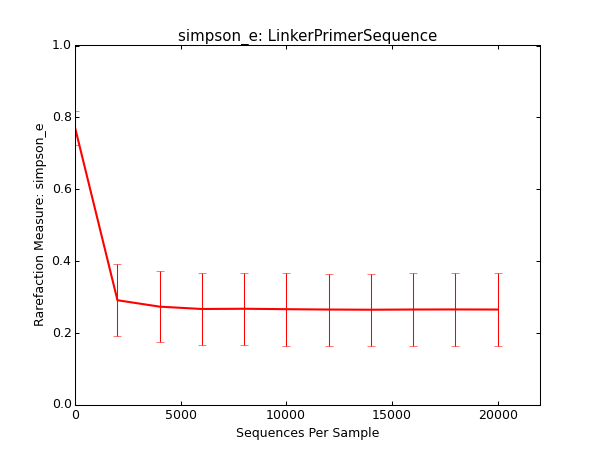

Supplement: Supplementary file 1 [file pathogens-15-00658-s001.zip › pathogens-4357168-File S1/Alpha_Diversity/Rarefaction_Plots/average_plots/simpson_eLinkerPrimerSequence.png]

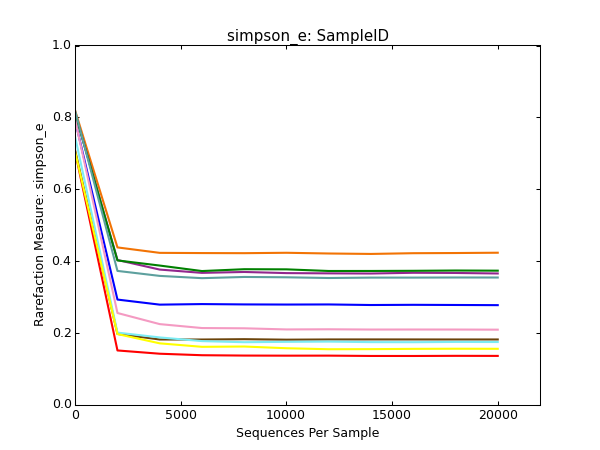

Supplement: Supplementary file 1 [file pathogens-15-00658-s001.zip › pathogens-4357168-File S1/Alpha_Diversity/Rarefaction_Plots/average_plots/simpson_eSampleID.png]

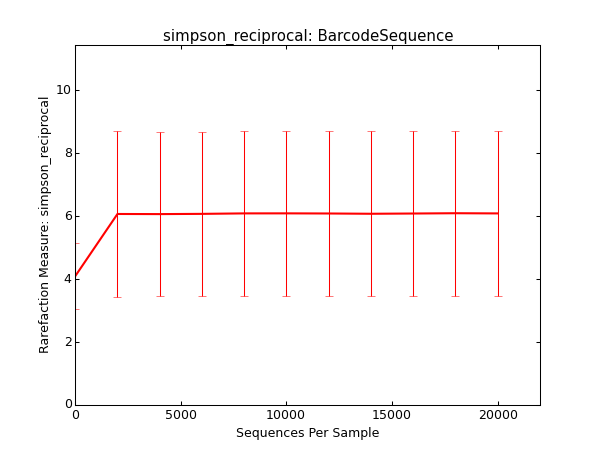

Supplement: Supplementary file 1 [file pathogens-15-00658-s001.zip › pathogens-4357168-File S1/Alpha_Diversity/Rarefaction_Plots/average_plots/simpson_reciprocalBarcodeSequence.png]

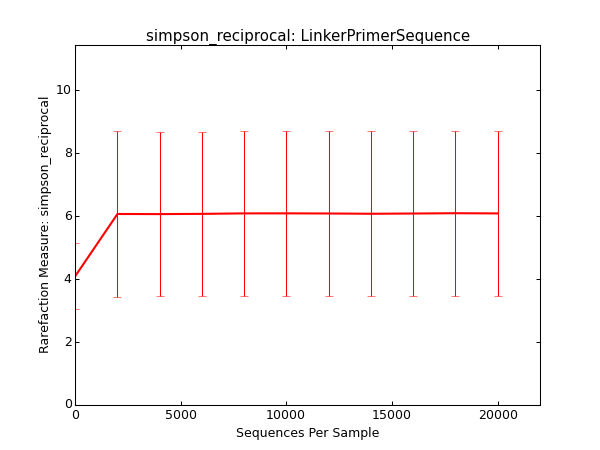

Supplement: Supplementary file 1 [file pathogens-15-00658-s001.zip › pathogens-4357168-File S1/Alpha_Diversity/Rarefaction_Plots/average_plots/simpson_reciprocalLinkerPrimerSequence.png]

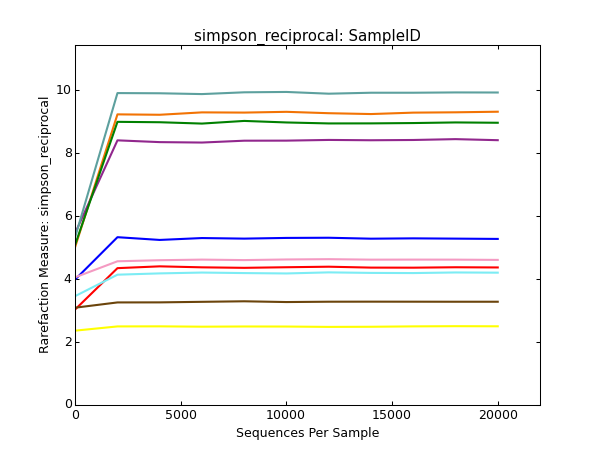

Supplement: Supplementary file 1 [file pathogens-15-00658-s001.zip › pathogens-4357168-File S1/Alpha_Diversity/Rarefaction_Plots/average_plots/simpson_reciprocalSampleID.png]

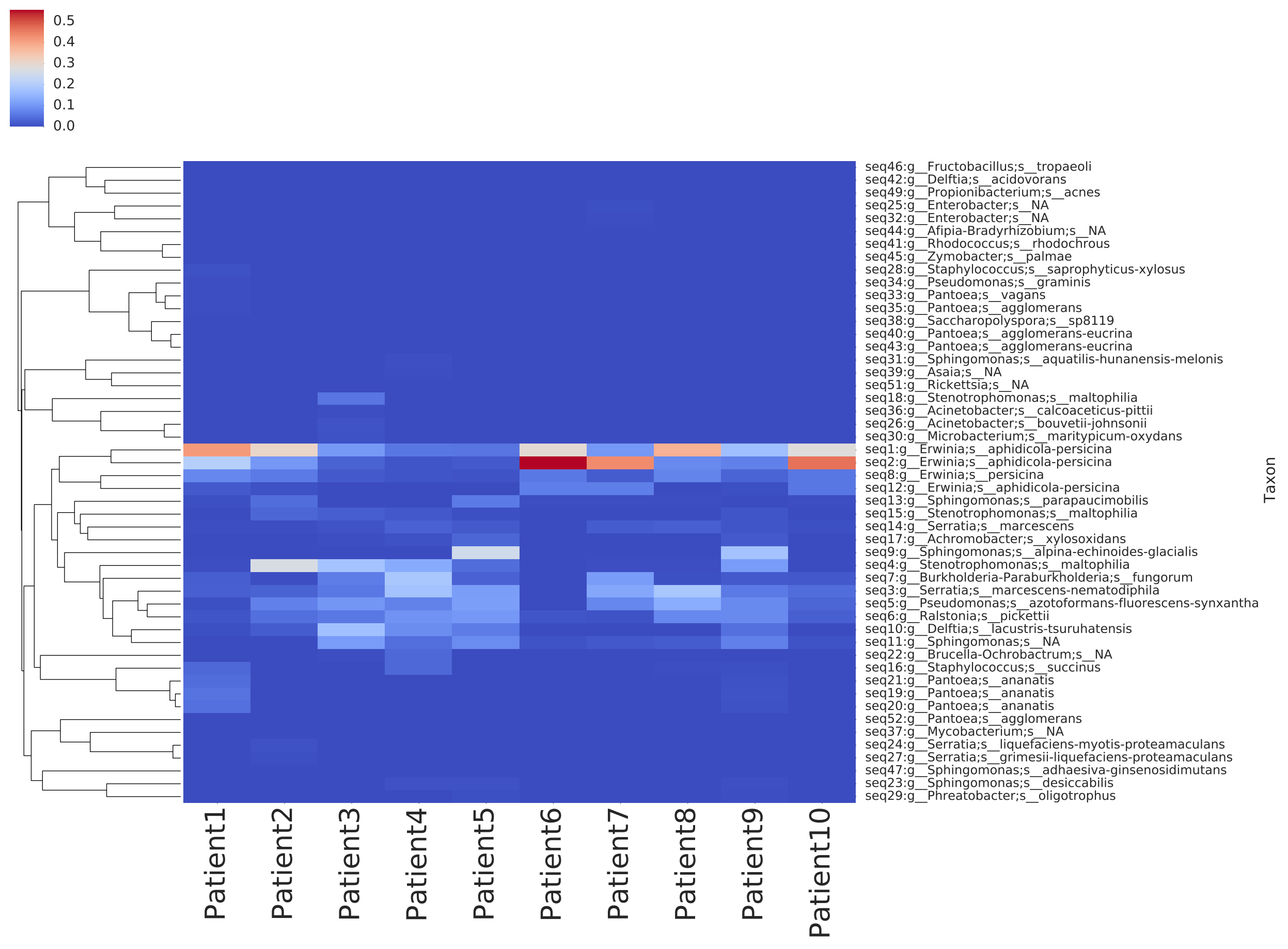

Supplement: Supplementary file 1 [file pathogens-15-00658-s001.zip › pathogens-4357168-File S1/ASV_Heatmap/Heatmap_without_SampleClustering.pdf]

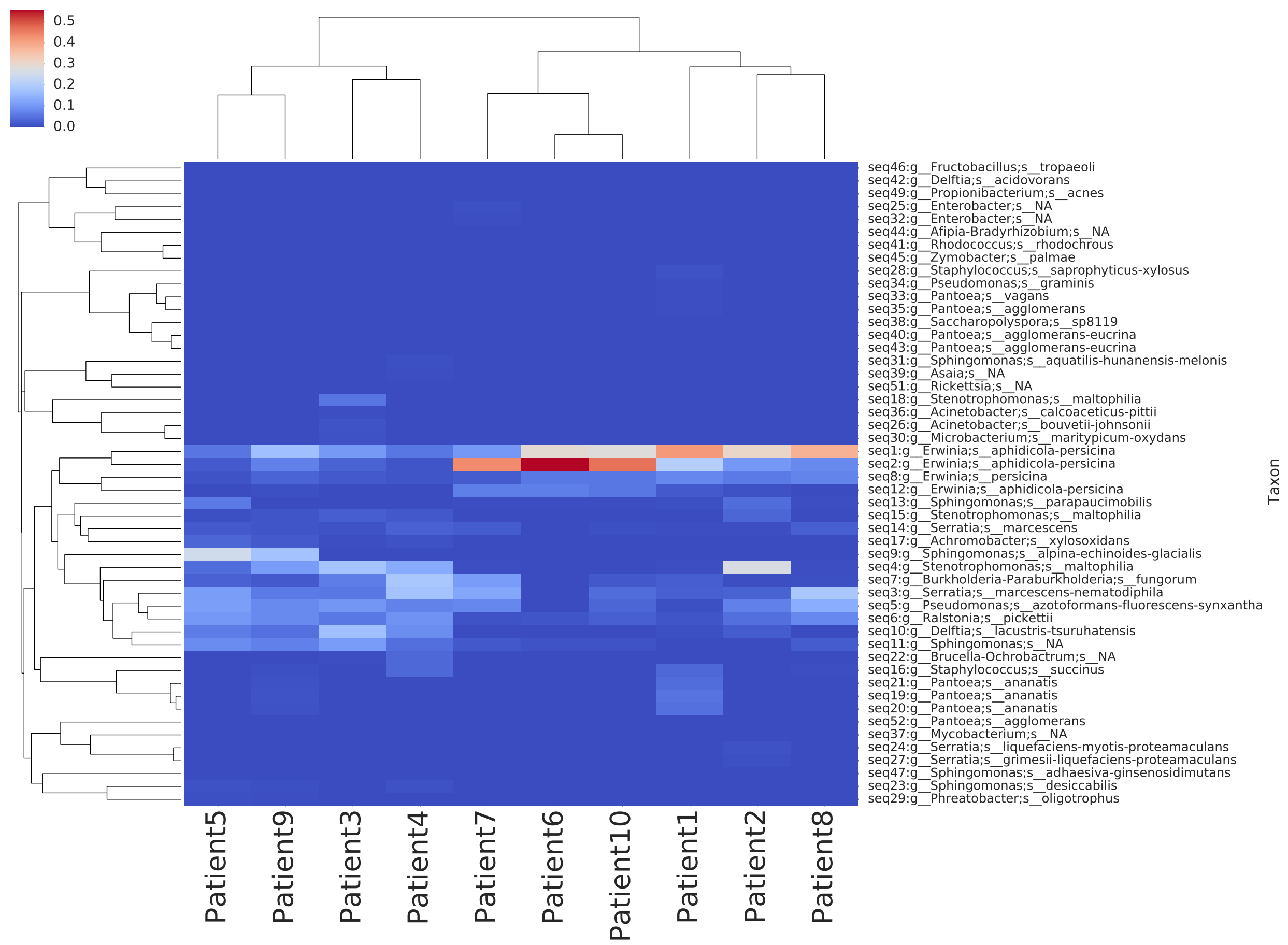

Supplement: Supplementary file 1 [file pathogens-15-00658-s001.zip › pathogens-4357168-File S1/ASV_Heatmap/Heatmap_with_SampleClustering.pdf]

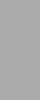

Supplement: Supplementary file 1 [file pathogens-15-00658-s001.zip › pathogens-4357168-File S1/Beta_Diversity_ASV/Bray_Curtis/emperor_required_resources/css/images/ui-bg_flat_0_aaaaaa_40x100.png]

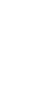

Supplement: Supplementary file 1 [file pathogens-15-00658-s001.zip › pathogens-4357168-File S1/Beta_Diversity_ASV/Bray_Curtis/emperor_required_resources/css/images/ui-bg_flat_75_ffffff_40x100.png]

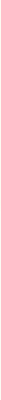

Supplement: Supplementary file 1 [file pathogens-15-00658-s001.zip › pathogens-4357168-File S1/Beta_Diversity_ASV/Bray_Curtis/emperor_required_resources/css/images/ui-bg_glass_55_fbf9ee_1x400.png]

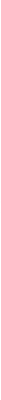

Supplement: Supplementary file 1 [file pathogens-15-00658-s001.zip › pathogens-4357168-File S1/Beta_Diversity_ASV/Bray_Curtis/emperor_required_resources/css/images/ui-bg_glass_65_ffffff_1x400.png]

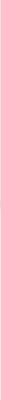

Supplement: Supplementary file 1 [file pathogens-15-00658-s001.zip › pathogens-4357168-File S1/Beta_Diversity_ASV/Bray_Curtis/emperor_required_resources/css/images/ui-bg_glass_75_dadada_1x400.png]

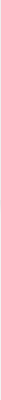

Supplement: Supplementary file 1 [file pathogens-15-00658-s001.zip › pathogens-4357168-File S1/Beta_Diversity_ASV/Bray_Curtis/emperor_required_resources/css/images/ui-bg_glass_75_e6e6e6_1x400.png]

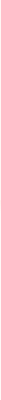

Supplement: Supplementary file 1 [file pathogens-15-00658-s001.zip › pathogens-4357168-File S1/Beta_Diversity_ASV/Bray_Curtis/emperor_required_resources/css/images/ui-bg_glass_95_fef1ec_1x400.png]

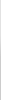

Supplement: Supplementary file 1 [file pathogens-15-00658-s001.zip › pathogens-4357168-File S1/Beta_Diversity_ASV/Bray_Curtis/emperor_required_resources/css/images/ui-bg_highlight-soft_75_cccccc_1x100.png]

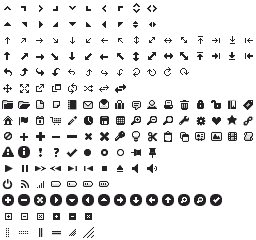

Supplement: Supplementary file 1 [file pathogens-15-00658-s001.zip › pathogens-4357168-File S1/Beta_Diversity_ASV/Bray_Curtis/emperor_required_resources/css/images/ui-icons_222222_256x240.png]

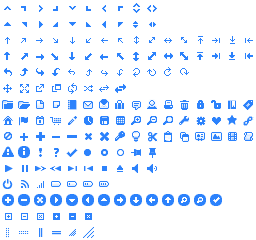

Supplement: Supplementary file 1 [file pathogens-15-00658-s001.zip › pathogens-4357168-File S1/Beta_Diversity_ASV/Bray_Curtis/emperor_required_resources/css/images/ui-icons_2e83ff_256x240.png]

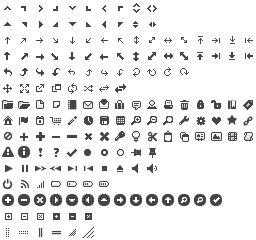

Supplement: Supplementary file 1 [file pathogens-15-00658-s001.zip › pathogens-4357168-File S1/Beta_Diversity_ASV/Bray_Curtis/emperor_required_resources/css/images/ui-icons_454545_256x240.png]

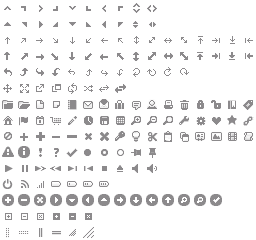

Supplement: Supplementary file 1 [file pathogens-15-00658-s001.zip › pathogens-4357168-File S1/Beta_Diversity_ASV/Bray_Curtis/emperor_required_resources/css/images/ui-icons_888888_256x240.png]

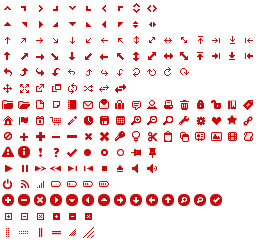

Supplement: Supplementary file 1 [file pathogens-15-00658-s001.zip › pathogens-4357168-File S1/Beta_Diversity_ASV/Bray_Curtis/emperor_required_resources/css/images/ui-icons_cd0a0a_256x240.png]

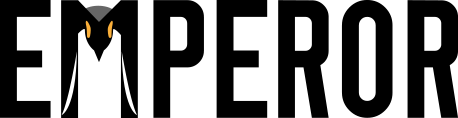

Supplement: Supplementary file 1 [file pathogens-15-00658-s001.zip › pathogens-4357168-File S1/Beta_Diversity_ASV/Bray_Curtis/emperor_required_resources/img/emperor.png]

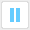

Supplement: Supplementary file 1 [file pathogens-15-00658-s001.zip › pathogens-4357168-File S1/Beta_Diversity_ASV/Bray_Curtis/emperor_required_resources/img/pause.png]

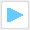

Supplement: Supplementary file 1 [file pathogens-15-00658-s001.zip › pathogens-4357168-File S1/Beta_Diversity_ASV/Bray_Curtis/emperor_required_resources/img/play.png]

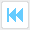

Supplement: Supplementary file 1 [file pathogens-15-00658-s001.zip › pathogens-4357168-File S1/Beta_Diversity_ASV/Bray_Curtis/emperor_required_resources/img/reset.png]
